# The Burden of Liver Cancer in Selected East Asian Countries (1990–2021) and Projections up to 2036: A Systematic Analysis of the Global Burden of Disease Study 2021

**DOI:** 10.3390/cancers18081272

**Published:** 2026-04-16

**Authors:** Tianhao Guo, Yu Zhao, Linyu Xu, Yumo Yuan, Yifan Hui, Tingting Zhou, Wenjian Zhu, Liu Li, Weixing Shen, Haibo Cheng, Xiaoyu Wu

**Affiliations:** 1 Jiangsu Collaborative Innovation Center of Traditional Chinese Medicine Prevention and Treatment of Tumor, The First Clinical Medical College, Nanjing University of Chinese Medicine, Nanjing 210023, China; guotianhao1996@163.com (T.G.);; 2 Department of Surgical Oncology, Affiliated Hospital of Nanjing University of Chinese Medicine (Jiangsu Province Hospital of Chinese Medicine), Nanjing 210029, China; 3 Institute of Health and Regimen, Jiangsu Open University, Nanjing 210036, China; 4 Department of General Surgery, Zhangjiagang TCM Hospital Affiliated to Nanjing University of Chinese Medicine, Zhangjiagang 215600, China; 5 School of Elderly Care Services and Management, Nanjing University of Chinese Medicine, Nanjing 210023, China; 6 Wangjing Hospital of China Academy of Chinese Medical Sciences, Beijing 100102, China; 7 Department of Oncology, Affiliated Hospital of Nanjing University of Chinese Medicine (Jiangsu Province Hospital of Chinese Medicine), Nanjing 210029, China

**Keywords:** liver cancer, disease burden, global burden of disease study, epidemiological study, decomposition analysis, joinpoint regression analysis, age–period–cohort analysis, prediction model

## Abstract

**Background**: Liver cancer remains a persistent global health challenge due to its high occurrence and fatality rates, critically impacting health outcomes and quality of life. The disease burden in East Asia, considering China, Japan, South Korea, and Mongolia, is among the highest globally. This study aims to examine the patterns and trends of liver cancer in selected East Asian countries from 1990 to 2021. **Methods**: This study employed data from the Global Burden of Disease Study 2021 pertaining to key burden metrics—prevalence, incidence, mortality, years of life lived with disability (YLDs), years of life lost (YLLs), and disability-adjusted life years (DALYs)—for selected East Asian countries from 1990 to 2021. Furthermore, the epidemiological trends and patterns were analyzed using decomposition analysis, Joinpoint regression, and age–period–cohort methods. Projections of the cancer burden through 2036 were generated using the Bayesian age–period–cohort model. **Results**: For all key indicators (incidence, prevalence, mortality, YLDs, YLLs, and DALYs), China had the highest values in both 1990 and 2021. The highest age-standardized rates for incidence (ASIR), mortality (ASMR), prevalence (ASPR), YLDs, YLLs, and DALYs (ASDR) were recorded in Mongolia. For all measured indicators of disease burden, the peak rates in each of the selected East Asian nations were consistently concentrated in the population aged 50 and above. Mongolia is forecasted to maintain the top-ranking ASIR values among the selected East Asian nations considered up to 2036. **Conclusions**: Over the past three decades, liver cancer has imposed a heavy disease burden on all selected East Asian nations, with rates being notably higher in the elderly population. These insights will provide policy-makers with evidence to guide public health strategies for disease prevention.

## Simple Summary

As a leading cause of cancer-related deaths globally, liver cancer critically impacts health outcomes, with the disease burden being most prominent in East Asia. This research assesses liver cancer burden across selected East Asian countries (China, Japan, South Korea, and Mongolia) from 1990 to 2021 using data from the Global Burden of Disease Study 2021. China had the highest values for prevalence, incidence, mortality, years of life lived with disability, years of life lost, and disability-adjusted life years in both 1990 and 2021. Mongolia had the highest age-standardized rates for prevalence, incidence, mortality, years of life lived with disability, years of life lost, and disability-adjusted life years in both 1990 and 2021. For three decades, liver cancer has exacted a heavy toll across East Asia—especially in the aging population. This article provides information to support public health policy-making in East Asia.

## 1. Introduction

In 2022, liver cancer ranked as the sixth most common cancer globally, constituting 4.3% of all cancer cases [1]. Liver cancer accounted for 7.8% of global cancer fatalities, making it the third leading cause of cancer-related deaths [1]. A consistently elevated incidence rate is observed annually for liver cancer [2], particularly in China [3]. China has approximately 42.4% of global liver cancer cases, and its liver cancer mortality is about 42.1% of the worldwide total [4]. Liver cancer is an escalating public health challenge; without intervention, the global incidence of liver cancer is projected to increase from approximately 870,000 cases in 2022 to about 1.52 million by 2050, nearly double the number [4,5].

According to data from the United Nations, Department of Economic and Social Affairs, Population Division (2022) (https://population.un.org/wpp (accessed on 15 July 2025)), the East Asian countries collectively account for about 22% of the global population and 24% of the world’s GDP. Common characteristics among the selected countries include geographical location, cultural influences, and dietary and lifestyle habits.

Liver cancer registries remain insufficient, and epidemiological reports are largely limited in comparing the selected East Asian countries. In Mongolia in particular, the insufficient cancer registration rate may be due to the vast area and sparse population or other specific reasons. The Global Burden of Disease Study 2021 (GBD 2021) provided a systematic assessment of mortality rates and years of life lost, with a total of 288 specific causes of death examined [6]. The analysis, spanning the period from 1990 to 2021, categorized data by age, sex, and location. It encompassed a comprehensive dataset from 204 countries and regions, inclusive of 811 subnational areas [6,7].

The aim of this study was to gain valuable insight into the overall burden of liver cancer in selected East Asian countries, providing a comprehensive assessment of disease burden by analyzing data from the GBD 2021. These insights will provide policy-makers with evidence to guide public health strategies for disease prevention. For each country, we first analyzed temporal trends in disease burden, followed by a cross-sectional comparison of the selected nations.

## 2. Methods

### 2.1. Design, Population, and Setting

Our study was conducted to examine patterns and trends in liver cancer across selected East Asian countries from 1990 to 2021. The analysis evaluated prevalence, incidence, mortality, years lived with disability (YLDs), years of life lost (YLLs), and disability-adjusted life years (DALYs). In this study, absolute burden (comprising the above indicators) reflected the total magnitude of disease burden, which is influenced by both population size and age structure. The study population included both sexes and all ages, with cases and population denominators covering the full age range (0–100+ years), according to the GBD age groups. The Bayesian age–period–cohort (BAPC) model was also applied to forecast the disease burden up to 2036.

### 2.2. Data Source

To obtain data spanning the years 1990 to 2021, we used the Global Health Data Exchange GBD Results Tool (https://vizhub.healthdata.org/gbd-results/ (accessed on 16 September 2024)). All age-standardized and age-specific rates, along with data incorporating 95% uncertainty intervals (UIs), were accessible through the GBD platform. The GBD study synthesizes data from multiple sources—such as cancer registries, vital registration systems, surveys, and scientific studies—around the world. This integration helps compensate for regions where cancer registries are sparse or incomplete. The GBD study uses sophisticated statistical modeling to quantify the burden of disease in regions where direct health data are unavailable or incomplete. These models leverage available partial data and covariates to produce more reliable incidence, prevalence, and mortality estimates. Variability in the quality of data between countries and over time is a potential limitation of the GBD estimates, impacting their comparability. In regions with limited or incomplete health data, reliance on statistical estimates and modeling may result in increased uncertainty. Disparities in data collection techniques and reporting criteria present a challenge, potentially affecting the reliability and uniformity of the findings. When interpreting the findings of the GBD study, it is advisable to take these factors into account.

Within the GBD 2021, the burden of disease attributed to specific behavioral and metabolic risk factors—such as dietary risks, high body mass index (BMI), smoking, and alcohol use—was estimated by employing the comparative risk assessment (CRA) framework. The CRA quantifies the fraction of disease burden that would be averted if population exposures were shifted from their observed distributions to a specified counterfactual distribution as the theoretical minimum risk exposure level (TMREL). For each risk–outcome pair, the GBD 2021 combined (1) exposure distributions by age, sex, location, and year; (2) relative risk (RR) functions describing the exposure–outcome relationship drawn from systematic reviews and meta-analyses; and (3) the TMREL to compute the population attributable fraction (PAF). PAFs were then applied to cause-specific deaths and YLDs to estimate attributable deaths and YLDs; attributable DALYs were obtained by summing attributable YLLs and attributable YLDs. All estimates incorporate uncertainty from exposures, RRs, and outcome estimates through Monte Carlo sampling to produce 95% UIs. Developed as a composite measure, the Socio-Demographic Index (SDI) captures the social and economic determinants of health for specific geographic areas [7]. The SDI is calculated as the geometric mean of three normalized indicators (ranging from 0 to 1): the total fertility rate among those under 25 (TFU25), the mean years of education for individuals aged 15 and above (EDU15+), and per capita lag-distributed income (LDI) [8]. In the GBD 2021, SDI values were multiplied by 100 after calculation to express the index on a 0–100 scale [8]. For each study year, all GBD geographies were ranked by their SDI values. Annual rankings were then used to assign geographies to SDI quintiles: the lowest 20% were classified as “Low SDI” and the next 20% as “Low–middle SDI,” continuing in this manner up to the highest 20%, which were classified as “High SDI.” This dynamic, rank-based approach accommodates changes in development over time [9].

From the GBD 2021, we obtained data on liver cancer burden (prevalence, incidence, mortality, YLDs, YLLs, and DALYs) for all age groups; the data encompassed the selected East Asian countries mentioned above and spanned the years 1990 to 2021.

### 2.3. Definition

All cancer cases with the International Classification of Diseases (ICD)-9 code 155 or ICD-10 code C22 were categorized as liver cancer for the purposes of this study. Consistent and precise identification of liver cancer cases across various data sources and geographies was achieved by applying the ICD coding framework, thereby supporting reliable burden assessment.

### 2.4. Ethics Statement

This study was granted an exemption from ethical review by the Ethics Committee under the category of “Previous data research”. This determination was based on the use of publicly accessible, aggregated population-level data, which does not involve individual patient records. This includes the use of existing data, records, discarded pathological specimens, or examination samples, which are either public resources or anonymized by the researcher to ensure that the subjects cannot be identified either directly or indirectly. As a result, the Ethics Committee of Affiliated Hospital of Nanjing University of Chinese Medicine granted this study an exemption from ethical review.

### 2.5. Data Analysis

Key statistical approaches employed in the GBD 2021—including Cause of Death Ensemble Modeling, Bayesian Meta-Regression, and uncertainty quantification—are detailed in the corresponding publication on methodology [6]. The trends for key burden metrics (prevalence, incidence, mortality, YLDs, YLLs, and DALYs) over the period 1990–2021 were examined across yearly, sex-specific, and age-stratified dimensions.

The average annual percent change (AAPC) and annual percentage change (APC), along with their 95% confidence intervals (CIs), were calculated to evaluate trends in all age-standardized rates (ASRs), including those for incidence (ASIR), mortality (ASMR), prevalence (ASPR), YLDs, YLLs, and DALYs (ASDR) [10]. The above ASRs remove the confounding effect of population aging by applying a standard age distribution, thereby reflecting the underlying epidemiological transition. We performed these calculations using Joinpoint regression analysis (Joinpoint Regression Program, Version 5.1.0) [11]. In accordance with the GBD World Population Age Standard, all ASRs were calculated.

The age–period–cohort model is based on a generalized linear model with a Poisson distribution [12,13]. To address the inherent identifiability issue arising from the linear dependence between age, period, and cohort, we applied the default constraints implemented in the Epi package. Specifically, the effects were centered using sum-to-zero constraints, and the linear component was allocated to the age effect, such that period and cohort effects were presented as non-linear deviations from the overall trend. In addition, we fitted penalized spline (second-difference) versions of the model available in Epi to obtain smoothed estimates; smoothing parameters were selected via generalized cross-validation and the Akaike information criterion.

Decomposition analysis was performed using the method originally developed by Kitagawa (1955) [14] and later generalized by Das Gupta (1978, 1991) [15,16]. This approach partitions the total change in prevalence/incidence/mortality between two time points (1990 and 2021) into three distinct components: population growth, population aging, and epidemiological change [17]. The contribution of each component is calculated by simultaneously considering the population size and prevalence/incidence/mortality rates at both the start and end of the study period, thereby avoiding order-dependence [17]. The results are expressed as absolute contributions (change in prevalence/incidence/mortality) and as percentages relative to the total change [18]. Percentages exceeding 100% occur when the positive contribution of one component is partially offset by negative contributions from other components. This analytical framework provides a comprehensive understanding of the drivers underlying temporal trends in disease burden.

To forecast the cancer burden up to 2036, the analysis was implemented in R using the BAPC and INLA packages to apply the BAPC model [19,20,21]. The analytical environment comprised R software (version 4.4.1) and the Zstats v1.0 package (www.zstats.net (accessed on 16 September 2024)). Within this environment, a two-tailed *p*-value below 0.05 was set as the threshold for statistical significance.

## 3. Results

### 3.1. Overall Burden

In both 1990 and 2021, Mongolia consistently reported the highest ASPR, ASIR, ASMR, age-standardized YLDs rate, age-standardized YLLs rate, and ASDR among the selected East Asian nations (Tables S1–S6).

When compared externally, the collective ASPR, ASIR, ASMR, and age-standardized YLDs rates of these selected countries surpassed both the global and Asian averages in 1990. By 2021, the selected countries continued to exceed these external benchmarks (Tables S1–S4).

In 1990, the age-standardized YLLs rate and ASDR of the selected East Asian countries surpassed global and regional (Asian) benchmarks. In 2021, the age-standardized YLLs rate and ASDR of the selected East Asian countries, with the exception of Japan, exceeded both global and Asian levels. Conversely, Japan’s age-standardized YLLs rate and ASDR were lower than both the global and the Asian levels (Tables S5 and S6).

### 3.2. Descriptive Analysis from Age Perspective

The age distribution of liver cancer prevalence and incidence varied by sex across East Asia in 2021. China and Mongolia exhibited early age peaks (males 50–54 and 55–59; females 65–69 and 60–64), whereas Japan and South Korea showed progressively later peaks (males 75–79 and 60–64; females 85–89 and 75–79) (Figures S1 and S2).

The age distribution of liver cancer mortality in 2021 exhibited distinct patterns between sexes across East Asia. China and Mongolia exhibited early age peaks (males 65–69 and 55–59; females 65–69 and 60–64), whereas Japan and South Korea showed progressively later peaks (males 80–84 and 60–64; females 85–89 and 80–84) (Figure S3).

The age patterns of YLDs due to liver cancer in 2021 differed markedly by sex across East Asia. China and Mongolia exhibited early age peaks (males 50–54 and 55–59; females 65–69 and 60–64), whereas Japan and South Korea showed progressively later peaks (males 75–79 and 60–64; females 85–89 and 75–79) (Figure S4).

The age distribution of YLLs and DALYs in 2021 revealed consistent sex-based disparities across East Asia. China and Mongolia exhibited early age peaks (males 50–54 and 55–59; females 65–69 and 60–64), whereas Japan and South Korea showed progressively later peaks (males 70–74 and 60–64; females 80–84 and 75–79) (Figures S5 and S6).

**Figure 1:**
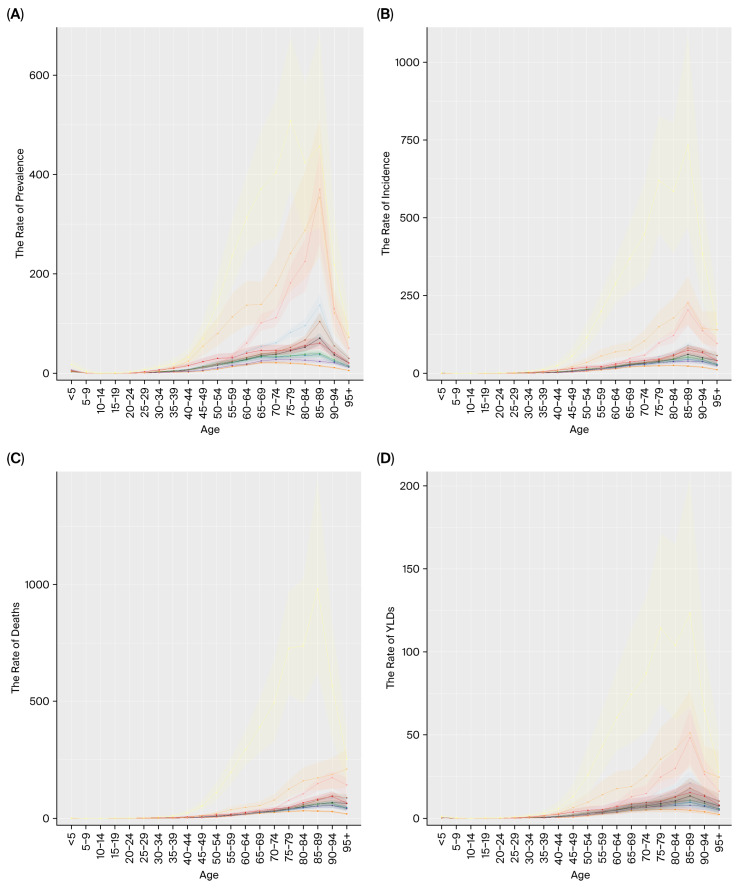

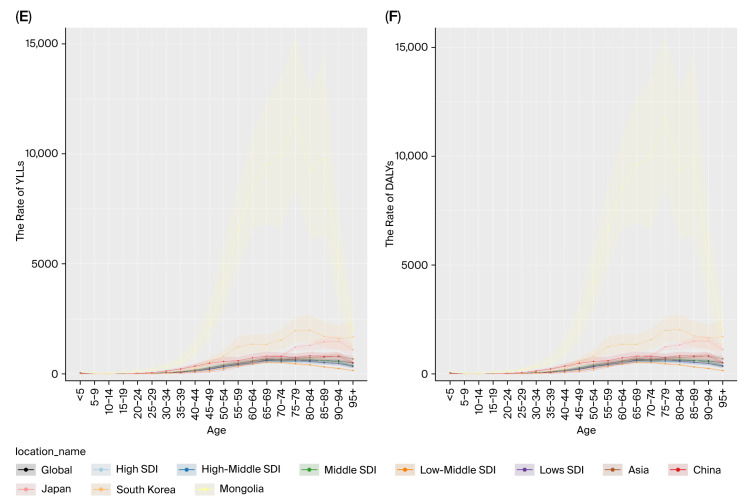
Age-specific rates in selected East Asian countries. (**A**) Age-specific prevalence rate, (**B**) age-specific incidence rate, (**C**) age-specific deaths rate, (**D**) age-specific YLDs rate, (**E**) age-specific YLLs rate, and (**F**) age-specific DALYs rate.

In 2021, the highest prevalence rates of liver cancer in China, Japan, and South Korea were consistently observed in the 85–89 age group. By contrast, the peak prevalence occurred in earlier age groups in Mongolia (75–79 years) (Figure 1A).

The 85–89 age group recorded the highest liver cancer incidence rate in the East Asian countries studied in 2021 (Figure 1B).

The age distribution of peak liver cancer mortality in 2021 revealed three distinct patterns across the region: it occurred in the 90–94 age group for China and Japan, at 95 years and older for South Korea, and in the 85–89 age group for Mongolia (Figure 1C).

The highest YLDs rate in 2021 was observed in the 85–89 age group across four East Asian countries (China, Japan, South Korea, Mongolia) (Figure 1D).

The age patterns of peak YLLs and DALYs rates in 2021 varied across the region. Japan’s highest rate was in the 90–94 age group. In China and South Korea, the peak occurred earlier (80–84 years). In Mongolia, the mortality peak appeared earliest, specifically observed in individuals aged 75 to 79 years (Figure 1E,F).

### 3.3. Descriptive Analysis from Period Perspective

**Figure 2:**
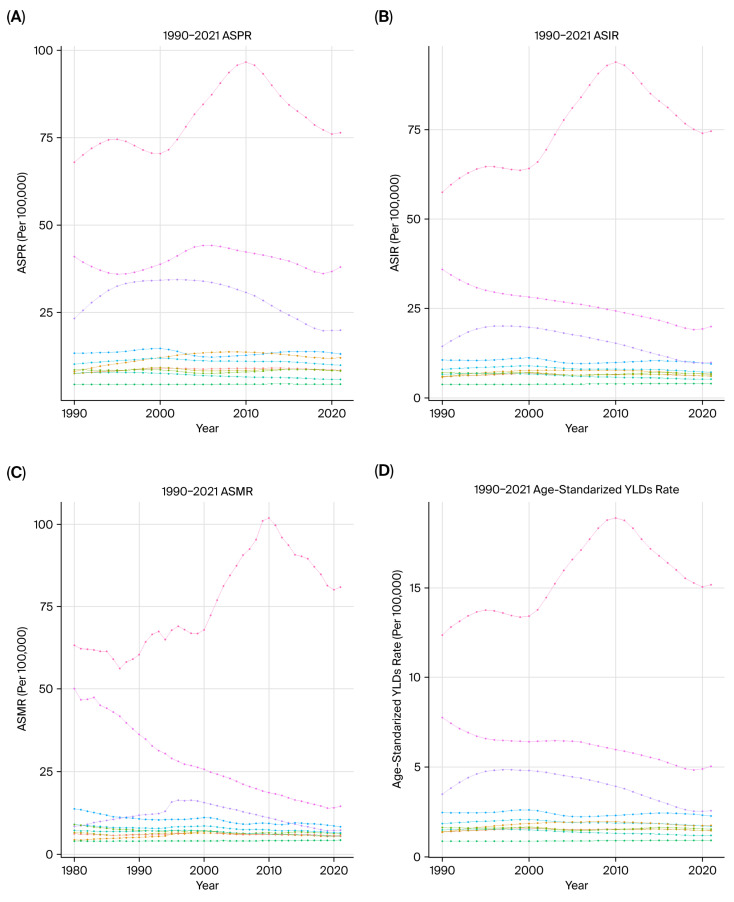

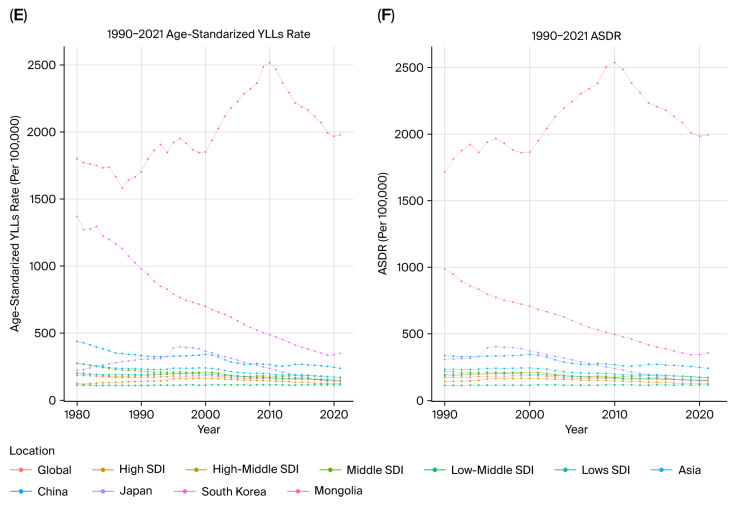
Time trends in selected East Asian countries. (**A**) ASPR, (**B**) ASIR, (**C**) ASMR, (**D**) age-standardized YLDs rate, (**E**) age-standardized YLLs rate, and (**F**) ASDR.

From 1990 to 2021, the ASPR, ASIR, ASMR, age-standardized YLDs rate, age-standardized YLLs rate, and ASDR shared an overall decreasing trend in selected East Asian countries, excluding Mongolia (Figure 2). In Mongolia, these six metrics fluctuated but showed an overall increase, consistently ranking the highest among the selected East Asian countries (Figure 2). Throughout the same period, all these six age‑standardized rates in each of these countries remained above the global levels (Figure 2).

From 1990 to 2021, the ASPR for females in China exhibited a generally fluctuating decline, whereas for males, it displayed an oscillating upward trend. In Japan, South Korea, the ASPR for both females and males followed an overall oscillating downward trajectory during the same period (Figure S7). Between 1990 and 2021, the ASIR, ASMR, age-standardized YLDs rate, age-standardized YLLs rate, and ASDR for both females and males in China, Japan, and South Korea displayed a fluctuating yet overall downward trend (Figures S8–S12). Conversely, during the same period, the ASPR, ASIR, ASMR, age-standardized YLDs rate, and ASDR for both females and males in Mongolia exhibited a generally oscillating upward trend (Figures S7–S12).

**Figure 3:**
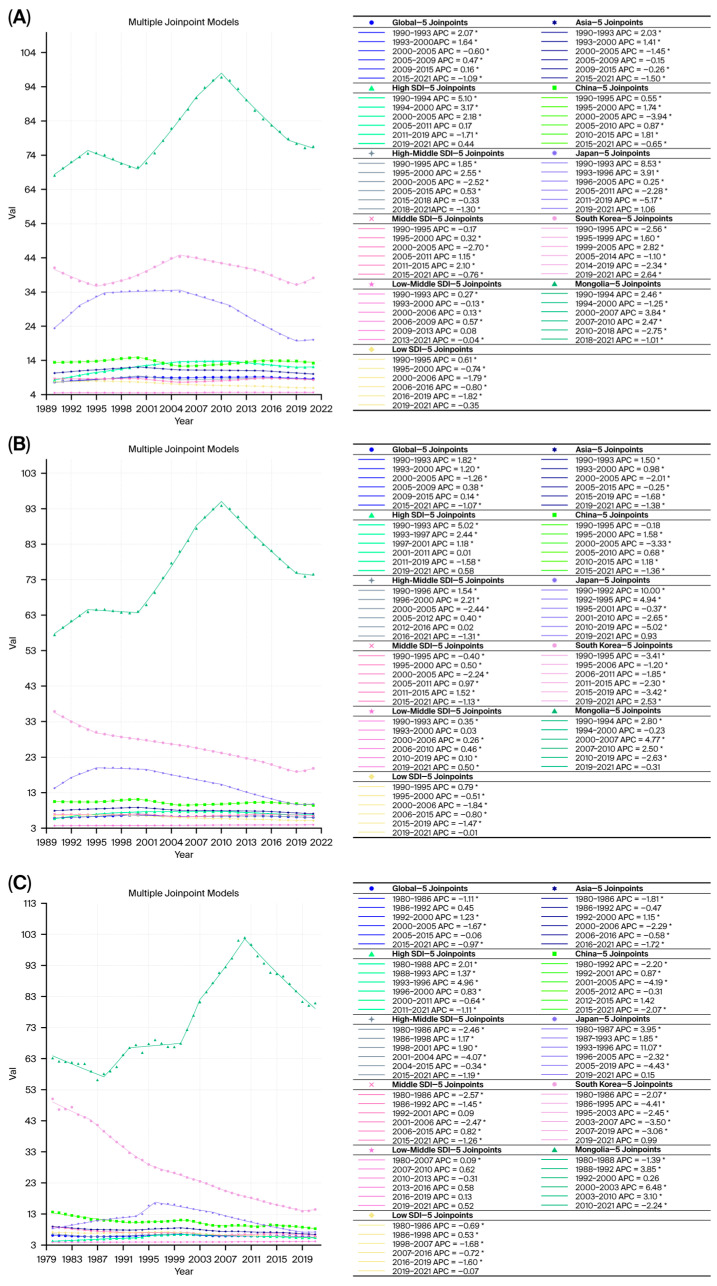

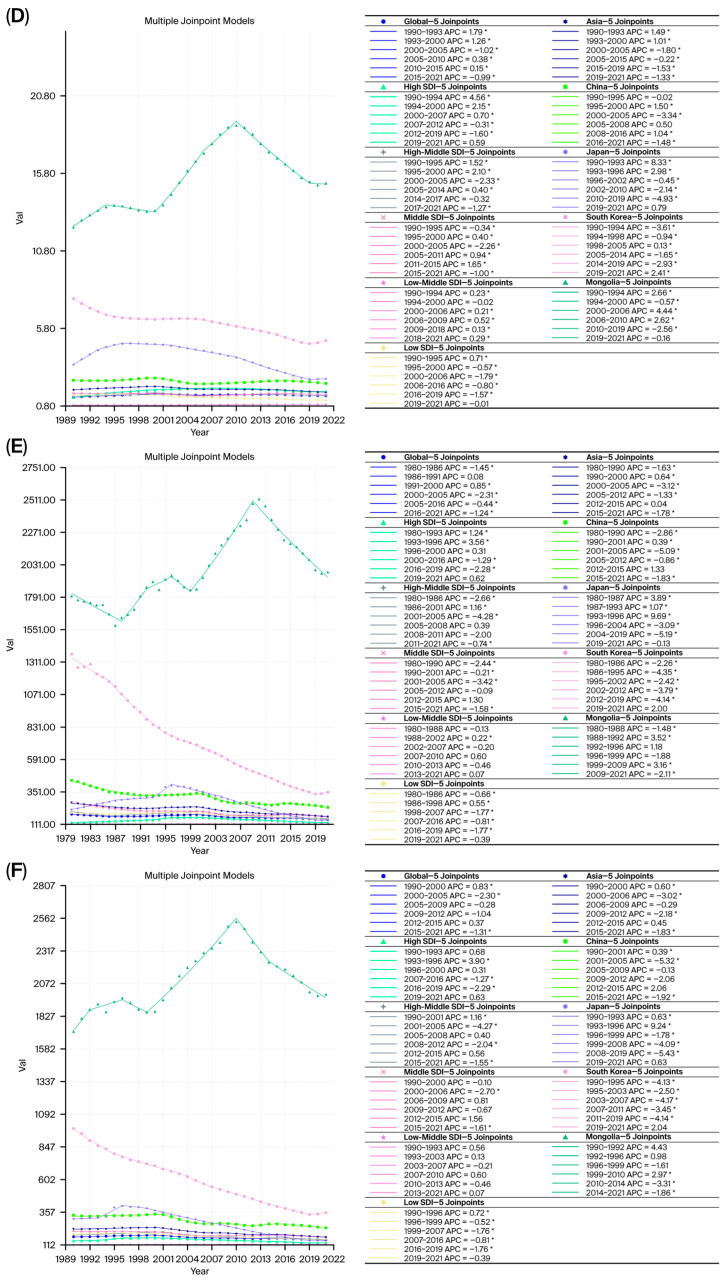
Joinpoint regression analysis of the age-standardized rates in selected East Asian countries from 1990 to 2021. (**A**) ASPR, (**B**) ASIR, (**C**) ASMR, (**D**) age-standardized YLDs rate, (**E**) age-standardized YLLs rate, and (**F**) ASDR. * Indicates that the Annual Percent Change (APC) is significantly different from zero at the alpha = 0.05 level.

Joinpoint regression analysis showed that the AAPCs for ASPR, ASIR, ASMR, age-standardized YLDs rate, age-standardized YLLs rate, and ASDR in China, Japan, and South Korea remained below the global averages (Figure 3 and Table 1). Conversely, the AAPCs for ASIR, ASMR, age-standardized YLDs rate, age-standardized YLLs rate, and ASDR in Mongolia exceeded the global levels (Figure 3 and Table 1).

### 3.4. Age–Period–Cohort Analysis

The prevalence rates in China and South Korea decreased from birth to 10 years old and increased sharply between ages 10 and 80, followed by a gradual decline after 80 years (Figure S13A,C). In Japan, the prevalence rate similarly declined until age 10 and then rose rapidly between ages 10 and 85, followed by a gradual decline in the population over 85 years of age (Figure S13B). In Mongolia, the prevalence rate declined from 0 to 10 years, rose steeply from ages 10 to 70, and exhibited a slow decline after 70 years (Figure S13D).

In China and Japan, the incidence and deaths rates decreased from birth to age 10 and rose sharply between ages 10 and 90, followed by a gradual decline after age 90 (Figures S14A,B and S15A,B). South Korea’s incidence and deaths rates similarly declined from 0 to 10 years but surged between 10 and 100 years (Figures S14C and S15C). In Mongolia, the incidence and deaths rates decreased from birth to 10 years old, increased rapidly between ages 10 and 85, and gradually declined beyond 85 years (Figures S14D and S15D).

### 3.5. Decomposition Analysis

**Figure 4:**
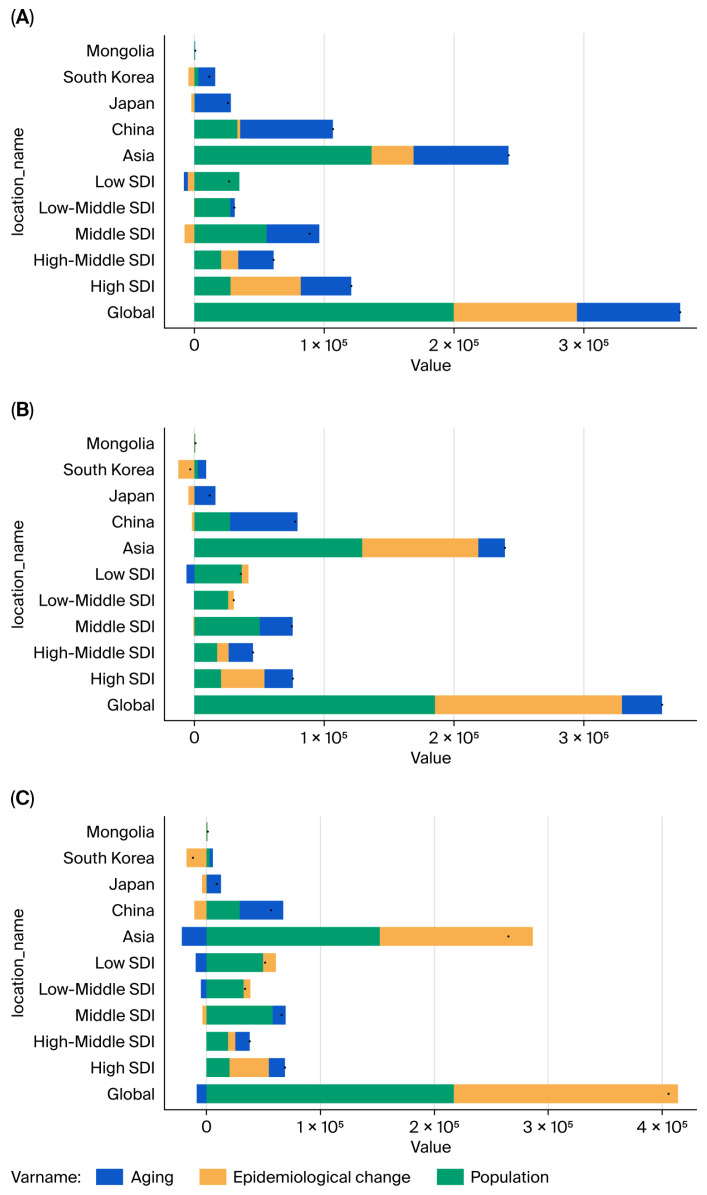
Decomposition analysis: (**A**) prevalence; (**B**) incidence; (**C**) mortality.

The prevalence rates in selected East Asian countries, China (66.81%), Japan (105.78%), and South Korea (110.82%) and excluding Mongolia (18.99%), are driven by aging and exceed both global (21.28%) and Asian (30.19%) averages (Figure 4A).

Similarly, the incidence rates are aging-related and surpass global (8.57%) and Asian (8.56%) averages in China (66.69%) and Japan (134.52%), but not in Mongolia (6.80%) or South Korea (−203.71%) (Figure 4B).

Mortality rates, influenced by aging, also exceed global (−2.10%) and Asian (−8.12%) averages in China (67.18%), Japan (136.89%), and Mongolia (−1.83%), but not in South Korea (−21.80%) (Figure 4C).

### 3.6. BAPC Prediction

**Figure 5:**
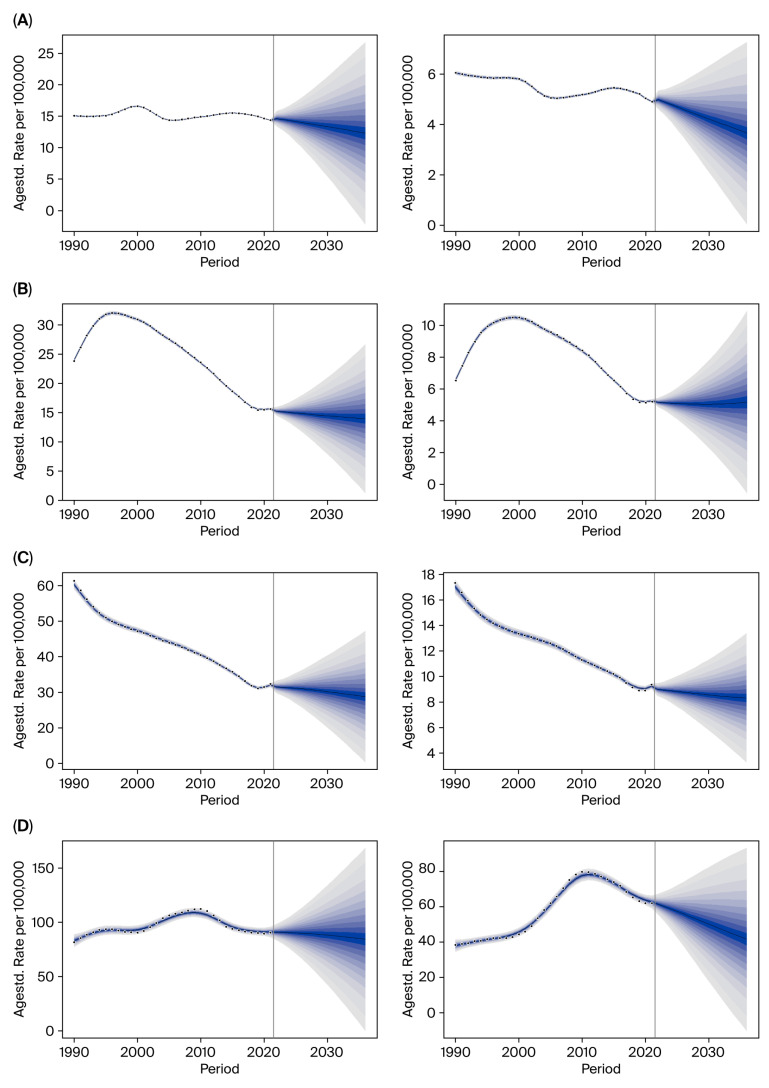
Bayesian age–period–cohort prediction of ASIR. (**A**) China, (**B**) Japan, (**C**) South Korea, and (**D**) Mongolia. Agestd.—age-standardized. The regions of different colors in the figure represent the uncertainty intervals of predicted values ranging from 5% to 95% with intervals of 5%.

We utilized the BAPC model to project trends up to 2036. Compared with 2021, the ASIRs of China, Japan, South Korea, and Mongolia are predicted to decrease by 2036; with Mongolia’s disease burden remaining the heaviest (Figure 5 and Table S7).

## 4. Discussion

Our results highlight distinct epidemiological trends and regional profiles, an understanding of which is essential for shaping public health strategies and ensuring efficient resource allocation. Our analysis reveals that among the selected East Asian nations, China reported the highest figures for incidence, prevalence, mortality, YLLs, YLDs, and DALYs in both 1990 and 2021. Mongolia showed the highest ASIR, ASMR, ASPR, age-standardized YLDs rate, age-standardized YLLs rate, and ASDR among the selected East Asian countries in both 1990 and 2021. Liver cancer imposes a heavy health burden on these East Asian nations, with China and Mongolia being notably affected. Based on projections generated by the BAPC model extending to 2036, Mongolia is forecasted to maintain the top-ranking ASIR among the selected East Asian nations considered. Furthermore, individuals aged 50 and above in this region consistently demonstrated the highest burden across all measured metrics—prevalence, incidence, mortality, YLDs, YLLs, and DALYs—regarding both absolute and standardized values. These results collectively underscore the disproportionately heavy burden of disease affecting the elderly population.

Our study acknowledges several limitations. The accuracy of the cancer burden estimates is heavily contingent upon the quality and completeness of the underlying data sources. While advanced statistical modeling was employed to partially address data gaps, inherent uncertainties remain. High-SDI regions often have a higher reported incidence due to a combination of more frequent detection, older populations, and lifestyle factors that increase true risk. Low/middle-SDI regions may be experiencing rapid increases in risk exposures and lifestyle changes while lacking full screening and treatment infrastructure, leading to rising incidence and sometimes stable or increasing mortality. When primary data are scarce, GBD modeled estimates typically have wider UIs. Important local drivers (nutrition, conflict-related injuries, and access to care) may not be adequately captured by covariates borrowed from other countries. Consequently, both point estimates and UIs should be interpreted cautiously. Although Mongolia has made improvements in its capture of health information, its sparse, geographically dispersed population and nomadic tradition reduce the representativeness of facility-based surveillance. A harsh climate and mining-related environmental exposures may produce local risk patterns that are poorly reflected by regional covariates. These factors can cause deviations between the true burden and modeled estimates. Additionally, our methodological approach does not incorporate all potential sources of uncertainty. Specifically, variability associated with the selection of covariates within the models remains unquantified. Attributing burden to specific risk factors often depends on modeled exposure estimates and relative risks derived from other populations; causal attribution may therefore be uncertain. The absence of reliable stage-at-diagnosis and treatment data may limit the ability to determine whether higher mortality arises from higher incidence, later stages at diagnosis, or poorer access to effective treatment. The ASR for primary liver cancer mortality showed general consistency across the three data sources examined. The most precise alignment, however, was observed between estimates from the World Health Organization (WHO) and those from the Global Cancer Observatory (GCO) [22]. The divergent trends in the WHO and GBD data for ten countries suggest potential inconsistencies, raising questions about real disease patterns in these regions [22].

Gastrointestinal cancers are responsible for almost one-third of global cancer deaths, with liver cancer comprising a substantial portion [23], and the burden of digestive cancers is increasing [24]. Liver cancer has been globally recognized as a major contributor to cancer mortality, with the heaviest burden in 2019 concentrated in several countries across East Asia, South Asia, West Africa, and North Africa [25,26]. It ranked as the second leading cause of cancer-related mortality and DALYs among men, accounting for 572,000 deaths and 15.2 million DALYs, following lung cancer and preceding stomach cancer in 2017 [27]. Regarding incidence rates, hepatitis B virus (HBV) infection is identified as the primary underlying cause of liver cancer, followed by hepatitis C virus (HCV) infection, alcohol consumption, and nonalcoholic steatohepatitis [26]. HBV infection remains a major public health challenge worldwide. In low- and low–middle-SDI regions, it contributes to a rising burden of cirrhosis and other chronic liver diseases. Conversely, in high-SDI regions, it is associated with increasing ASIR [28]. The ASPR for HCV-related liver cancer has shown a global upward trend. This rise has been particularly pronounced in low–middle-SDI regions over the last ten years [29]. According to the results of the GBD 2019, gastrointestinal and liver cancers continued to rank high in China [30]. Between 1990 and 2019, liver cancer in China declined at an estimated annual rate of −5.17 (95% CI: −6.00, −4.33) [31]. The leading historical cause of liver cancer in China has been chronic infection with HBV [32]. The introduction of routine hepatitis B vaccination for infants in the late 1990s has resulted in a measurable reduction in liver cancer burden among younger generations, but the incidence remains high among older cohorts infected perinatally or in early childhood [33]. Dietary aflatoxin exposure contributed to liver cancer in some rural regions in the past, but its impact has diminished with improvements in food safety. A high HCV prevalence is the primary driver of Mongolia’s liver cancer burden [34]; it has also had a major impact on older cohorts in Japan [35]. Japan’s lower incidence and mortality due to liver cancer reflect well-established surveillance and early-detection systems for liver disease and cancer. However, alcohol consumption and metabolic risk factors—such as obesity, diabetes, and nonalcoholic fatty liver disease—are increasing and could counteract some of the gains achieved through HBV control in China and Japan [35].

Over the past decade, global absolute deaths and ASDR associated with non-alcoholic steatohepatitis-related liver cancer (NALC) have increased, showing pronounced disparities across age, sex, and regions. Population aging, growth, and metabolism-related factors have been the primary contributors to the rise in global NALC deaths [36]. While age-standardized rates for alcohol-related liver cancer and cirrhosis have decreased, the absolute disease burden has increased—a trend that is projected to continue [37]. A high BMI and diet-related risks are increasingly prominent contributors to the disease burden in many middle-income countries. These shifts reflect rapid changes in food systems, urbanization, and lifestyle. Greater intake of pickled, smoked, or preserved foods in some East Asian regions can increase exposure to carcinogens (nitrosamines), potentially raising cancer risks. Targeted screening is very important, considering that liver cancer mainly occurs in the elderly population. Simultaneously, enhancing rehabilitation services and long-term care for this patient population is essential. This approach aims to enhance the quality of life for patients while alleviating the broader societal and healthcare burden associated with the disease [38].

East Asian populations exhibit a unique profile of cancer-associated genes compared with other regions [39,40,41]. The existing literature reveals a scarcity of research focused on epidemiological trends in liver cancer across the East Asian countries considered in this study. Health disparities also warrant attention: even when effective therapies exist, their diffusion into clinical practice in lower-resource settings is often delayed or absent. Such barriers help explain why, in countries such as Mongolia, high mortality and DALYs burdens persist despite the availability of newer systemic therapies in high-income countries. These observations underscore the need to strengthen health systems and equity-oriented drug access policies to translate therapeutic advances into measurable reductions in disease burden [42].

## 5. Conclusions

In conclusion, liver cancer has imposed a persistent disease burden on East Asian countries. This trend has been particularly evident in East Asian countries over the last thirty years, with older adults being disproportionately affected. Given the multifaceted nature of liver cancer development, research efforts should be directed toward two primary goals: elucidating its underlying causes and developing improved treatment modalities. Public health initiatives must prioritize improving early detection and prevention for liver cancer. The concurrent optimization of healthcare resource distribution across different regions and rigorous implementation of effective environmental health policies are equally critical. Further research on liver cancer epidemiology among Asian populations is needed, with a particular emphasis on tailored management and targeted prevention and control strategies that address differences across regions, genders, and age groups.

## Figures and Tables

**Table 1:** AAPC from 1990 to 2021 at the global, regional, and selected East Asian countries levels.

Location	ASPR (95% CI)	ASIR (95% CI)	ASMR (95% CI)	Age-Standardized YLDs Rate (95% CI)	Age-Standardized YLLs Rate (95% CI)	ASDR (95% CI)
Global	0.35 (0.31, 0.39)	0.11 (0.07, 0.16)	−0.22 (−0.36, −0.08)	0.18 (0.13, 0.23)	−0.55 (−0.68, −0.42)	−0.46 (−0.67, −0.26)
SDI						
High SDI	1.22 (1.09, 1.34)	0.57 (0.47, 0.67)	0.52 (0.36, 0.68)	0.77 (0.67, 0.87)	−0.01 (−0.19, 0.18)	−0.42 (−0.73, −0.12)
High–middle SDI	0.32 (0.26, 0.37)	0.07 (0.02, 0.12)	−0.46 (−0.72, −0.20)	0.13 (0.08, 0.18)	−0.72 (−0.99, −0.46)	−0.63 (−0.88, −0.37)
Middle SDI	−0.05 (−0.15, 0.05)	−0.19 (−0.24, −0.14)	−0.84 (−1.00, −0.68)	−0.16 (−0.24, −0.09)	−1.15 (−1.34, −0.96)	−0.72 (−0.97, −0.47)
Low–middle SDI	0.08 (0.04, 0.12)	0.21 (0.18, 0.24)	0.15 (0.12, 0.18)	0.18 (0.15, 0.21)	0.04 (−0.02, 0.11)	0.08 (0.02, 0.14)
Low SDI	−0.83 (−0.91, −0.74)	−0.74 (−0.80, −0.68)	−0.60 (−0.66, −0.54)	−0.74 (−0.82, −0.66)	−0.65 (−0.72, −0.59)	−0.80 (−0.87, −0.74)
Asia	−0.09 (−0.13, −0.05)	−0.36 (−0.40, −0.32)	−0.75 (−0.94, −0.56)	−0.28 (−0.32. −0.25)	−1.11 (−1.34, −0.88)	−0.95 (−1.31, −0.60)
China	0.02 (−0.11, 0.15)	−0.31 (−0.39, −0.23)	−1.14 (−1.47, −0.82)	−0.23 (−0.33, −0.13)	−1.42 (−1.75, −1.09)	−0.96 (−1.36, −0.56)
Japan	−0.51 (−0.69, −0.33)	−1.20 (−1.36, −1.04)	−0.51 (−0.82, −0.21)	−1.00 (−1.20, −0.80)	−1.08 (−1.42, −0.74)	−2.38 (−2.58, −2.19)
South Korea	−0.21 (−0.33, −0.09)	−1.86 (−1.94, −1.77)	−2.95 (−3.15, −2.74)	−1.37 (−1.45, −1.29)	−3.24 (−3.45, −3.04)	−3.24 (−3.45, −3.04)
Mongolia	0.34 (0.13, 0.56)	0.81 (0.63, 1.00)	0.52 (0.01, 1.04)	0.64 (0.51, 0.77)	0.16 (−0.14, 0.46)	0.43 (−0.24, 1.11)

AAPC: average annual percent change, ASPR: age-standardized prevalence rate, ASIR: age-standardized incidence rate, ASMR: age-standardized mortality rate, ASDR: age-standardized disability-adjusted life years rate.

## Data Availability

The GBD 2021 data resources are available online through the Global Health Data Exchange (GHDx) query tool (http://ghdx.healthdata.org/gbd-results-tool (accessed on 16 September 2024)). Data used for the analyses are publicly available from the Institute of Health Metrics and Evaluation (http://www.healthdata.org/; http://ghdx.healthdata.org/gbd-results-tool (accessed on 16 September 2024)).

## Supplementary Materials

The following supporting information can be downloaded at: https://www.mdpi.com/article/10.3390/cancers18081272/s1, Figure S1: Age-specific prevalence numbers in selected East Asian countries. Figure S2: Age-specific incidence numbers in selected East Asian countries. Figure S3: Age-specific deaths numbers in selected East Asian countries. Figure S4: Age-specific YLDs numbers in selected East Asian countries. Figure S5: Age-specific YLLs numbers in selected East Asian countries. Figure S6: Age-specific DALYs numbers in selected East Asian countries. Figure S7: Trends in the all-age prevalence number and rate by sex from 1990 to 2021. Figure S8: Trends in the all-age incidence number and rate by sex from 1990 to 2021. Figure S9: Trends in the all-age deaths number and rate by sex from 1990 to 2021. Figure S10: Trends in the all-age YLDs number and rate by sex from 1990 to 2021. Figure S11: Trends in the all-age YLLs number and rate by sex from 1990 to 2021. Figure S12: Trends in the all-age DALYs number and rate by sex from 1990 to 2021. Figure S13: Age–period–cohort analysis of prevalence rates: (A) China, (B) Japan, (C) South Korea, and (D) Mongolia. Figure S14: Age–period–cohort analysis of incidence rates: (A) China, (B) Japan, (C) South Korea, and (D) Mongolia. Figure S15: Age–period–cohort analysis of deaths rates: (A) China, (B) Japan, (C) South Korea, and (D) Mongolia. Table S1: Prevalence from 1990 to 2021 at the global, regional, and selected East Asian countries levels. Table S2: Incidence from 1990 to 2021 at the global, regional, and selected East Asian countries levels. Table S3: Deaths from 1990 to 2021 at the global, regional, and selected East Asian countries levels. Table S4: YLDs (Years Lived with Disability) from 1990 to 2021 at the global, regional, and selected East Asian countries levels. Table S5: YLLs (Years of Life Lost) from 1990 to 2021 at the global, regional, and selected East Asian countries levels. Table S6: DALYs (Disability-Adjusted Life Years) from 1990 to 2021 at the global, regional, and selected East Asian country levels. Table S7: BAPC prediction ASIR in selected East Asian countries.

## Abbreviations

AAPC	average annual percent change
ASDR	age-standardized disability-adjusted life years rate
ASIR	age-standardized incidence rate
ASMR	age-standardized mortality rate
ASPR	age-standardized prevalence rate
BAPC	Bayesian age–period–cohort
DALYs	disability-adjusted life years
GBD	Global Burden of Disease
HBV	Hepatitis B virus
HCV	Hepatitis C virus
ICD	International Classification of Diseases
YLDs	years lived with disability
YLLs	years of life lost

## References

[1] Bray F., Laversanne M., Sung H., Ferlay J., Siegel R.L., Soerjomataram I., Jemal A. (2024). Global cancer statistics 2022: GLOBOCAN estimates of incidence and mortality worldwide for 36 cancers in 185 countries. CA Cancer J. Clin..

[2] Siegel R.L., Miller K.D., Fuchs H.E., Jemal A. (2022). Cancer statistics, 2022. CA Cancer J. Clin..

[3] Xia C., Dong X., Li H., Cao M., Sun D., He S., Yang F., Yan X., Zhang S., Li N. (2022). Cancer statistics in China and United States, 2022: Profiles, trends, and determinants. Chin. Med. J..

[4] Chan S.L., Sun H.C., Xu Y., Zeng H., El-Serag H.B., Lee J.M., Schwartz M.E., Finn R.S., Seong J., Wang X.W. (2025). The Lancet Commission on addressing the global hepatocellular carcinoma burden: Comprehensive strategies from prevention to treatment. Lancet.

[5] Miller K.D., Nogueira L., Devasia T., Mariotto A.B., Yabroff K.R., Jemal A., Kramer J., Siegel R.L. (2022). Cancer treatment and survivorship statistics, 2022. CA Cancer J. Clin..

[6] (2024). GBD 2021 Causes of Death Collaborators. Global burden of 288 causes of death and life expectancy decomposition in 204 countries and territories and 811 subnational locations, 1990–2021: A systematic analysis for the Global Burden of Disease Study 2021. Lancet.

[7] GBD 2021 Demographics Collaborators (2024). Global age-sex-specific mortality, life expectancy, and population estimates in 204 countries and territories and 811 subnational locations, 1950–2021, and the impact of the COVID-19 pandemic: A comprehensive demographic analysis for the Global Burden of Disease Study 2021. Lancet.

[8] GBD 2021 Diseases and Injuries Collaborators (2024). Global incidence, prevalence, years lived with disability (YLDs), disability-adjusted life-years (DALYs), and healthy life expectancy (HALE) for 371 diseases and injuries in 204 countries and territories and 811 subnational locations, 1990–2021: A systematic analysis for the Global Burden of Disease Study 2021. Lancet.

[9] (2017). GBD 2016 Mortality Collaborators. Global, regional, and national under-5 mortality, adult mortality, age-specific mortality, and life expectancy, 1970–2016: A systematic analysis for the Global Burden of Disease Study 2016. Lancet.

[10] Kim H.J., Fay M.P., Feuer E.J. (2017). Improved confidence interval for average annual percent change in trend analysis. Stat. Med..

[11] Kim H.J., Fay M.P., Feuer E.J., Midthune D.N. (2000). Permutation tests for joinpoint regression with applications to cancer rates. Stat. Med..

[12] Holford T.R. (1983). The estimation of age, period and cohort effects for vital rates. Biometrics.

[13] Holford T.R. (1992). Analysing the temporal effects of age, period and cohort. Stat. Methods Med. Res..

[14] Kitagawa E.M. (1955). Components of a difference between two rates. J. Am. Stat. Assoc..

[15] Das Gupta P. (1978). A general method of decomposing a difference between two rates into several components. Demography.

[16] Das Gupta P. (1991). Decomposition of the difference between two rates and its consistency when more than two populations are involved. Math. Popul. Stud..

[17] Andreev E.M., Shkolnikov V.M., Begun A.Z. (2002). Algorithm for decomposition of differences between aggregate demographic measures. Demogr. Res..

[18] Cheng X., Tan L., Gao Y., Yang Y., Schwebel D.C., Hu G. (2019). A new method to attribute differences in total deaths between groups to population size, age structure and age-specific mortality rate. PLoS ONE.

[19] Rue H., Martino S., Chopin N. (2009). Approximate Bayesian inference for latent Gaussian models by using integrated nested Laplace approximations. J. R. Stat. Soc. Ser. B.

[20] Riebler A., Held L. (2010). The analysis of heterogeneous time trends in multivariate age-period-cohort models. Biostatistics.

[21] Knoll M., Furkel J., Debus J., Abdollahi A., Karch A., Stock C. (2020). An R package for an integrated evaluation of statistical approaches to cancer incidence projection. BMC Med. Res. Methodol..

[22] Li C., He W.Q. (2022). Comparison of primary liver cancer mortality estimates from World Health Organization, global burden disease and global cancer observatory. Liver Int..

[23] Danpanichkul P., Suparan K., Tothanarungroj P., Dejvajara D., Rakwong K., Pang Y., Barba R., Thongpiya J., Fallon M.B., Harnois D. (2024). Epidemiology of gastrointestinal cancers: A systematic analysis from the Global Burden of Disease Study 2021. Gut.

[24] Yu Z., Bai X., Zhou R., Ruan G., Guo M., Han W., Jiang S., Yang H. (2024). Differences in the incidence and mortality of digestive cancer between Global Cancer Observatory 2020 and Global Burden of Disease 2019. Int. J. Cancer.

[25] Akinyemiju T., Abera S., Ahmed M., Alam N., Alemayohu M.A., Allen C., Al-Raddadi R., Alvis-Guzman N., Amoako Y., Artaman A. (2017). The Burden of Primary Liver Cancer and Underlying Etiologies From 1990 to 2015 at the Global, Regional, and National Level: Results From the Global Burden of Disease Study 2015. JAMA Oncol..

[26] Xing Q.Q., Li J.M., Dong X., Zeng D.Y., Chen Z.J., Lin X.Y., Pan J.S. (2022). Socioeconomics and attributable etiology of primary liver cancer, 1990–2019. World J. Gastroenterol..

[27] Fitzmaurice C., Abate D., Abbasi N., Abbastabar H., Abd-Allah F., Abdel-Rahman O., Abdelalim A., Abdoli A., Abdollahpour I., Abdulle A.S.M. (2019). Global, Regional, and National Cancer Incidence, Mortality, Years of Life Lost, Years Lived With Disability, and Disability-Adjusted Life-Years for 29 Cancer Groups, 1990 to 2017: A Systematic Analysis for the Global Burden of Disease Study. JAMA Oncol..

[28] Cao G., Jing W., Liu J., Liu M. (2022). Countdown on hepatitis B elimination by 2030: The global burden of liver disease related to hepatitis B and association with socioeconomic status. Hepatol. Int..

[29] Jing W., Liu J., Liu M. (2020). Global Trends and Regional Differences in Hepatitis C Virus Infection Prevalence and Implications for Prevention—Worldwide, 1990–2017. China CDC Wkly..

[30] Tang X., Wang P., Huang S., Peng J., Zhang W., Shi X., Shi L., Zhong X., Lyu M., Zhou X. (2024). Trend of gastrointestinal and liver diseases in China: Results of the Global Burden of Disease Study, 2019. Chin. Med. J..

[31] Cao G., Liu J., Liu M. (2022). Trends in mortality of liver disease due to hepatitis B in China from 1990 to 2019: Findings from the Global Burden of Disease Study. Chin. Med. J..

[32] Polaris Observatory Collaborators (2023). Global prevalence, cascade of care, and prophylaxis coverage of hepatitis B in 2022: A modelling study. Lancet Gastroenterol. Hepatol..

[33] Sun N., Shao X., Hou C., Wei X., Lin W., Yang Y., Chen L., Hon C., Zhu G., Sun J. (2026). Feasibility of eliminating adult hepatitis B in Guangdong by 2030: A modeling study. Infect. Dis. Model..

[34] Huang L., Chen X., Wang Z. (2025). Total burden of hepatitis B and C attributed to injecting drug use in 204 countries and territories from 1990 to 2021: Analyses based on the Global Burden of Disease Study 2021. Int. J. Infect. Dis..

[35] Lim R.Y., Koh B., Ng C.H., Kulkarni A.V., Liu K., Wijarnpreecha K., Kim B.K., Muthiah M.D., Lee S.W., Zheng M.H. (2025). Hepatocellular Carcinoma Surveillance and Survival in a Contemporary Asia-Pacific Cohort. JAMA Netw. Open.

[36] Liu C., Zhu S., Zhang J., Wu P., Wang X., Du S., Wang E., Kang Y., Song K., Yu J. (2023). Global, regional, and national burden of liver cancer due to non-alcoholic steatohepatitis, 1990–2019: A decomposition and age-period-cohort analysis. J. Gastroenterol..

[37] Liu Y., Sun Z., Wang Q., Wu K., Tang Z., Zhang B. (2023). Contribution of alcohol use to the global burden of cirrhosis and liver cancer from 1990 to 2019 and projections to 2044. Hepatol. Int..

[38] El-Shami K., Oeffinger K.C., Erb N.L., Willis A., Bretsch J.K., Pratt-Chapman M.L., Cannady R.S., Wong S.L., Rose J., Barbour A.L. (2015). American Cancer Society Colorectal Cancer Survivorship Care Guidelines. CA Cancer J. Clin..

[39] Chang J.S., Hsiao J.R., Chen C.H. (2017). ALDH2 polymorphism and alcohol-related cancers in Asians: A public health perspective. J. Biomed. Sci..

[40] Huang W., Skanderup A.J., Lee C.G. (2018). Advances in genomic hepatocellular carcinoma research. Gigascience.

[41] Tanaka Y., Ogawa E., Huang C.F., Toyoda H., Jun D.W., Tseng C.H., Hsu Y.C., Enomoto M., Takahashi H., Furusyo N. (2020). HCC risk post-SVR with DAAs in East Asians: Findings from the REAL-C cohort. Hepatol. Int..

[42] Kostadinov K., Popova-Sotirova I., Marinova Y., Musurlieva N., Iskrov G., Stefanov R. (2024). Availability and Access to Orphan Drugs for Rare Cancers in Bulgaria: Analysis of De-lays and Public Expenditures. Cancers.

