# Supplementary material for: The Burden of Liver Cancer in Selected East Asian Countries (1990–2021) and Projections up to 2036: A Systematic Analysis of the Global Burden of Disease Study 2021
Source: Cancers (Basel). 2026 Apr 16;18(8):1272. doi: 10.3390/cancers18081272 (PMC13115021; doi:10.3390/cancers18081272)
Supplement: Supplementary file 1 [file cancers-18-01272-s001.zip › cancers-4172898-supplementary/Table S1 Prevalence.pdf]

Table S1. Prevalence from 1990 to 2021 at the global, regional, and selected East Asian countries levels.

Location	1990 Prevalence cases (95% UI)			1990 Age-standardized rates per 100 000 people (95% UI)			2021 Prevalence cases (95% UI)			2021 Age-standardized rates per 100 000 people (95% UI)		
	Total	Male	Female	Total	Male	Female	Total	Male	Female	Total	Male	Female
Global	345913 (299827,376632)	236149 (207508,261581)	109764 (88716,123047)	7.76 (6.92,8.43)	11.03 (9.94,12.15)	4.69 (3.94,5.26)	739300 (673114,821948)	520567 (463953,600533)	218732 (195489,241403)	8.68 (7.9,9.67)	12.76 (11.43,14.69)	4.95 (4.44,5.46)
SDI												
High SDI	86780 (82354,90715)	62720 (59537,65947)	24060 (22120,25403)	8.33 (7.9,8.72)	13.18 (12.52,13.86)	4.13 (3.82,4.36)	235058 (213884,249190)	167962 (157184,177595)	67095 (56956,73602)	12.21 (11.3,12.86)	18.69 (17.62,19.75)	6.4 (5.64,6.89)
High-middle SDI	78560 (68889,88622)	56309 (47885,65305)	22251 (19235,25586)	7.67 (6.71,8.64)	11.63 (9.92,13.44)	4.16 (3.57,4.78)	155296 (132501,184367)	114478 (93529,142422)	40818 (34028,48566)	8.42 (7.21,9.96)	13.01 (10.66,16.06)	4.18 (3.51,4.92)
Middle SDI	113375 (96516,128049)	77919 (65226,90208)	35456 (28541,40733)	8.75 (7.62,9.83)	11.96 (10.3,13.89)	5.49 (4.61,6.28)	231125 (198437,276538)	167482 (138241,212129)	63643 (54557,75454)	8.42 (7.26,9.99)	12.41 (10.31,15.63)	4.62 (3.98,5.47)

Low-middle SDI	39209 (27885,45655)	23487 (17242,27658)	15722 (10313,18837)	4.62 (3.75,5.5)	5.53 (4.54,6.56)	3.67 (2.79,4.5)	74894 (66171,84279)	46710 (40497,54151)	28184 (24896,31509)	4.74 (4.23,5.31)	6.04 (5.27,6.98)	3.5 (3.12,3.91)
Low SDI	27804 (17093,34907)	15598 (10207,20142)	12205 (6551,16381)	7.8 (5.67,10.04)	9.18 (6.39,11.88)	6.38 (4.37,8.97)	42599 (31334,55640)	23713 (18077,31399)	18886 (13218,25170)	6.05 (4.93,7.74)	6.92 (5.54,9.05)	5.19 (4.06,6.62)
Asia	249363 (218279,274820)	177859 (155615,200716)	71504 (59019,81034)	10.37 (9.26,11.35)	14.67 (12.99,16.43)	5.99 (5.22,6.69)	508893 (450739,582108)	366893 (313702,443874)	142000 (123531,162865)	10.12 (8.95,11.55)	14.91 (12.83,17.89)	5.6 (4.86,6.43)
China	132779 (108924,155564)	95779 (78334,116603)	37000 (29627,44601)	13.51 (11.2,15.77)	18.94 (15.48,23.02)	7.86 (6.34,9.48)	265539 (212435,331149)	198826 (150846,266673)	66713 (51940,84332)	13.29 (10.75,16.41)	20 (15.38,26.47)	6.64 (5.19,8.32)
Japan	39819 (38106,41153)	29631 (28447,30746)	10188 (9369,10873)	23.46 (22.48,24.23)	37.99 (36.45,39.39)	10.99 (10.15,11.69)	72628 (61956,79894)	50091 (44916,54293)	22537 (16828,26670)	20.1 (17.94,21.65)	31.46 (28.66,33.92)	10.41 (8.47,11.76)
South Korea	13868 (10384,17505)	10523 (7734,13561)	3345 (2346,4267)	41.12 (31.06,51.59)	69.09 (51.65,87.1)	19.01 (13.41,24.39)	34966 (28721,42744)	26288 (21500,32186)	8678 (6610,11238)	38.16 (31.51,46.61)	61.39 (50.45,74.92)	17.54 (13.63,22.37)
Mongolia	993 (617,1383)	605 (386,854)	388 (235,551)	68.11 (46.19,93.58)	91.56 (62.99,129.05)	47.91 (32.02,67.34)	1924 (1474,2488)	1103 (820,1457)	821 (633,1069)	76.57 (58.82,97.89)	94.03 (70.22,122.08)	61.97 (47.49,81.35)
