# Supplementary material for: The Burden of Liver Cancer in Selected East Asian Countries (1990–2021) and Projections up to 2036: A Systematic Analysis of the Global Burden of Disease Study 2021
Source: Cancers (Basel). 2026 Apr 16;18(8):1272. doi: 10.3390/cancers18081272 (PMC13115021; doi:10.3390/cancers18081272)
Supplement: Supplementary file 1 [file cancers-18-01272-s001.zip › cancers-4172898-supplementary/Table S2 Incidence.pdf]

Table S2. Incidence between 1990 and 2021 at the global, regional, and selected East Asian countries levels.

**Location**	**1990 Incident cases (95% UI)**			**1990 Age-standardized rates per 100 000 people (95% UI)**			**2021 Incident cases (95% UI)**			**2021 Age-standardized rates per 100 000 people (95% UI)**		
	**Total**	**Male**	**Female**	**Total**	**Male**	**Female**	**Total**	**Male**	**Female**	**Total**	**Male**	**Female**
Global	244689 (224795,268549)	168995 (152011,186301)	75695 (67486,85265)	5.9 (5.43,6.48)	8.55 (7.73,9.39)	3.48 (3.1,3.93)	529202 (480339,593849)	364355 (321977,422727)	164848 (147932,181388)	6.15 (5.58,6.9)	8.98 (7.96,10.37)	3.6 (3.24,3.96)
SDI												
High SDI	62217 (58840,65523)	44286 (41881,46803)	17931 (16351,19109)	5.78 (5.46,6.09)	9.28 (8.78,9.81)	2.85 (2.61,3.04)	142546 (129644,151309)	98049 (91959,103275)	44497 (37511,48587)	6.92 (6.39,7.31)	10.48 (9.85,11.04)	3.78 (3.31,4.06)
High-middle SDI	60676 (53598,68150)	43280 (37203,49966)	17396 (15126,19903)	5.98 (5.3,6.71)	9.32 (8.06,10.69)	3.14 (2.73,3.59)	117674 (101084,138477)	84091 (69072,104580)	33583 (28116,39677)	6.1 (5.23,7.17)	9.42 (7.76,11.65)	3.13 (2.62,3.7)
Middle SDI	80014 (70274,90052)	55675 (47714,65110)	24339 (20883,27826)	7.03 (6.2,7.91)	9.66 (8.32,11.25)	4.4 (3.77,5.03)	179743 (155621,213998)	127749 (106051,160298)	51994 (44524,61584)	6.58 (5.72,7.79)	9.66 (8.04,12.03)	3.72 (3.18,4.4)

Low-middle SDI	25181 (21809,30490)	15630 (13571,18934)	9551 (7855,12114)	3.79 (3.23,4.68)	4.56 (3.94,5.6)	2.99 (2.39,3.91)	60181 (54565,66691)	37740 (33067,43523)	22441 (20112,24907)	4.05 (3.68,4.48)	5.21 (4.59,6)	2.96 (2.66,3.28)
Low SDI	16451 (12330,21501)	10031 (7068,13131)	6420 (4888,9020)	6.57 (4.83,8.85)	7.88 (5.52,10.57)	5.22 (3.79,7.63)	28787 (23444,36441)	16545 (13181,21522)	12242 (9884,15356)	5.24 (4.32,6.56)	6 (4.83,7.74)	4.49 (3.68,5.59)
Asia	174570 (157322,191617)	126144 (110976,142135)	48426 (42454,54372)	8 (7.25,8.78)	11.39 (10.06,12.74)	4.6 (4.02,5.16)	362876 (319312,418350)	256910 (217556,311455)	105966 (91692,121705)	7.19 (6.33,8.28)	10.51 (8.96,12.64)	4.08 (3.53,4.68)
China	96434 (80971,113769)	70209 (56752,85768)	26225 (20941,31755)	10.58 (8.94,12.43)	15.06 (12.2,18.24)	6.04 (4.82,7.28)	196637 (158273,243558)	143788 (108927,193831)	52848 (41045,67026)	9.52 (7.72,11.78)	14.34 (10.93,19.18)	4.89 (3.82,6.18)
Japan	24736 (23506,25434)	18447 (17796,18971)	6290 (5728,6628)	14.35 (13.62,14.76)	23.75 (22.84,24.45)	6.49 (5.93,6.82)	39163 (33437,42580)	25917 (23708,27594)	13246 (9696,15362)	9.89 (8.84,10.57)	15.48 (14.35,16.36)	5.16 (4.11,5.82)
South Korea	11349 (8533,14319)	8472 (6223,10866)	2877 (2034,3694)	35.88 (27.14,45.09)	61.09 (45.89,77.36)	17.22 (12.36,22.05)	18642 (15182,22884)	13768 (11199,16930)	4875 (3625,6196)	19.94 (16.27,24.49)	32.16 (26.37,39.34)	9.29 (7.02,11.76)
Mongolia	653 (464,898)	416 (286,590)	237 (165,336)	57.51 (40.35,79.73)	81.11 (55.81,116.5)	38.1 (26.33,54.08)	1710 (1316,2186)	955 (717,1243)	755 (580,979)	74.56 (57.16,94.46)	90.09 (66.7,116.53)	61.73 (47.1,81.02)
