# Supplementary material for: The Burden of Liver Cancer in Selected East Asian Countries (1990–2021) and Projections up to 2036: A Systematic Analysis of the Global Burden of Disease Study 2021
Source: Cancers (Basel). 2026 Apr 16;18(8):1272. doi: 10.3390/cancers18081272 (PMC13115021; doi:10.3390/cancers18081272)
Supplement: Supplementary file 1 [file cancers-18-01272-s001.zip › cancers-4172898-supplementary/Table S3 Deaths.pdf]

Table S3. Deaths from 1990 to 2021 at the global, regional, and selected East Asian countries levels.

Location	1990 Deaths cases (95% UI)			1990 Age-standardized rates per 100 000 people (95% UI)			2021 Deaths cases (95% UI)			2021 Age-standardized rates per 100 000 people (95% UI)		
	Total	Male	Female	Total	Male	Female	Total	Male	Female	Total	Male	Female
Global	238969 (218717,263037)	162843 (146106,179832)	76127 (67563,86272)	5.86 (5.38,6.46)	8.44 (7.61,9.3)	3.55 (3.15,4.03)	483875 (440400,540177)	324696 (288483,376834)	159179 (142936,175021)	5.65 (5.13,6.3)	8.1 (7.24,9.37)	3.46 (3.11,3.8)
SDI												
High SDI	56590 (53362,59788)	39563 (37277,41986)	17026 (15483,18195)	5.21 (4.9,5.52)	8.34 (7.84,8.85)	2.64 (2.41,2.82)	117889 (106689,125243)	78681 (73489,82878)	39208 (33001,42743)	5.51 (5.05,5.83)	8.26 (7.74,8.7)	3.13 (2.73,3.36)
High-middle SDI	60539 (53669,67803)	42543 (36614,49105)	17996 (15701,20574)	6.03 (5.37,6.75)	9.36 (8.14,10.74)	3.25 (2.84,3.71)	107715 (93069,125154)	74744 (61994,92627)	32971 (27744,38652)	5.53 (4.77,6.44)	8.43 (7,10.41)	3.01 (2.54,3.53)
Middle SDI	79627 (69973,89505)	54794 (46973,63913)	24833 (21351,28375)	7.24 (6.4,8.12)	9.86 (8.49,11.43)	4.65 (3.99,5.32)	166730 (145739,196163)	115819 (96600,144018)	50911 (43477,59908)	6.21 (5.43,7.26)	8.97 (7.5,11.08)	3.68 (3.14,4.33)

Low-middle SDI	25495 (21929,31214)	15749 (13643,19252)	9746 (7910,12539)	4 (3.41,4.96)	4.77 (4.12,5.89)	3.19 (2.53,4.21)	61938 (56193,68550)	38556 (33917,44388)	23382 (21016,25936)	4.26 (3.88,4.7)	5.46 (4.83,6.26)	3.15 (2.83,3.47)
Low SDI	16562 (12285,21841)	10098 (7091,13349)	6464 (4867,9220)	6.99 (5.13,9.48)	8.33 (5.84,11.21)	5.6 (4.04,8.29)	29329 (23876,37217)	16716 (13365,21725)	12613 (10219,15855)	5.6 (4.62,7.02)	6.35 (5.11,8.18)	4.87 (3.98,6.07)
Asia	168629 (151464,185189)	120635 (105742,135625)	47994 (41919,54131)	7.94 (7.19,8.74)	11.21 (9.89,12.53)	4.7 (4.09,5.27)	325803 (287401,375776)	225899 (191921,274849)	99904 (86615,114543)	6.52 (5.74,7.52)	9.43 (8.08,11.37)	3.85 (3.34,4.42)
China	94937 (79884,111527)	68304 (55235,83128)	26633 (21350,32258)	10.75 (9.12,12.61)	15.19 (12.32,18.36)	6.33 (5.08,7.64)	172068 (139621,212496)	122463 (93115,164816)	49605 (38617,62668)	8.35 (6.8,10.29)	12.4 (9.46,16.55)	4.57 (3.57,5.76)
Japan	20308 (19265,20828)	15093 (14570,15461)	5214 (4730,5460)	11.8 (11.16,12.12)	19.69 (18.91,20.18)	5.33 (4.84,5.58)	31123 (26459,33773)	20243 (18446,21324)	10879 (8022,12568)	7.29 (6.48,7.77)	11.55 (10.67,12.1)	3.78 (2.97,4.24)
South Korea	11042 (8305,13956)	8143 (5986,10415)	2899 (2066,3711)	36.2 (27.45,45.34)	61.84 (46.99,78)	17.92 (12.88,22.93)	13674 (11219,16835)	10015 (8220,12369)	3659 (2717,4616)	14.53 (11.97,17.79)	23.77 (19.67,29.24)	6.78 (5.16,8.51)
Mongolia	666 (468,924)	425 (293,610)	241 (168,345)	60.4 (42.21,84.08)	85.91 (58.82,123.97)	39.86 (27.6,57.23)	1781 (1373,2278)	974 (728,1268)	807 (619,1056)	80.89 (62.08,102.56)	96.13 (71.1,124.93)	68.28 (52.19,90.56)
