# Supplementary material for: The Burden of Liver Cancer in Selected East Asian Countries (1990–2021) and Projections up to 2036: A Systematic Analysis of the Global Burden of Disease Study 2021
Source: Cancers (Basel). 2026 Apr 16;18(8):1272. doi: 10.3390/cancers18081272 (PMC13115021; doi:10.3390/cancers18081272)
Supplement: Supplementary file 1 [file cancers-18-01272-s001.zip › cancers-4172898-supplementary/Table S4 YLDs.pdf]

Table S4. YLDs (Years Lived with Disability) from 1990 to 2021 at the global, regional, and selected East Asian countries levels.

Location	1990 YLDs cases (95% UI)			1990 Age-standardized rates per 100 000 people (95% UI)			2021 YLDs cases (95% UI)			2021 Age-standardized rates per 100 000 people (95% UI)		
	Total	Male	Female	Total	Male	Female	Total	Male	Female	Total	Male	Female
Global	58642 (41909,78638)	40234 (27970,54820)	18408 (13003,25075)	1.39 (0.99,1.85)	1.99 (1.39,2.72)	0.83 (0.58,1.13)	126500 (89518,167809)	87361 (61378,117693)	39139 (27583,52299)	1.47 (1.04,1.96)	2.15 (1.52,2.89)	0.86 (0.61,1.15)
SDI												
High SDI	14666 (10368,19063)	10401 (7374,13490)	4265 (2987,5686)	1.37 (0.97,1.78)	2.18 (1.55,2.83)	0.69 (0.49,0.92)	35318 (25402,46443)	24490 (17587,32004)	10828 (7551,14506)	1.76 (1.25,2.3)	2.66 (1.9,3.47)	0.95 (0.68,1.27)
High-middle SDI	14276 (9895,19346)	10171 (6877,13946)	4104 (2858,5592)	1.4 (0.97,1.9)	2.17 (1.47,2.96)	0.75 (0.52,1.02)	27843 (19566,38163)	19992 (13587,28460)	7851 (5423,10881)	1.46 (1.02,2)	2.25 (1.54,3.17)	0.75 (0.52,1.03)

Middle SDI	19274 (13539,26103)	13340 (9009,18442)	5934 (4174,8055)	1.64 (1.15,2.23)	2.24 (1.51,3.1)	1.03 (0.72,1.4)	42248 (28984,57820)	30075 (20347,42295)	12173 (8298,16446)	1.54 (1.06,2.1)	2.26 (1.53,3.16)	0.87 (0.6,1.18)
Low-middle SDI	6231 (4360,8645)	3833 (2681,5289)	2397 (1655,3426)	0.88 (0.61,1.25)	1.05 (0.73,1.48)	0.69 (0.47,1.05)	14003 (9785,18728)	8764 (6040,11743)	5239 (3653,6975)	0.93 (0.65,1.23)	1.19 (0.82,1.59)	0.68 (0.47,0.91)
Low SDI	4159 (2796,6245)	2466 (1594,3681)	1693 (1081,2667)	1.5 (0.97,2.33)	1.78 (1.12,2.71)	1.21 (0.77,2.04)	7025 (4845,10363)	3998 (2732,6004)	3027 (2055,4586)	1.2 (0.82,1.76)	1.37 (0.92,2.04)	1.03 (0.7,1.53)
Asia	41806 (29568,56065)	29985 (20552,40662)	11822 (8325,16022)	1.86 (1.31,2.5)	2.63 (1.81,3.59)	1.08 (0.76,1.47)	86473 (61001,117319)	61320 (42702,84569)	25153 (17331,34262)	1.71 (1.21,2.31)	2.49 (1.74,3.43)	0.97 (0.67,1.32)
China	22981 (15783,31663)	16647 (10901,23757)	6334 (4255,8812)	2.47 (1.69,3.4)	3.49 (2.3,5.01)	1.43 (0.96,1.99)	46603 (31434,65156)	34142 (22296,50853)	12461 (8158,17953)	2.27 (1.53,3.15)	3.4 (2.25,5.04)	1.17 (0.77,1.69)
Japan	5980 (4291,7788)	4418 (3133,5729)	1561 (1112,2046)	3.48 (2.5,4.54)	5.67 (4.03,7.35)	1.63 (1.16,2.15)	9712 (6879,12793)	6490 (4612,8572)	3222 (2199,4410)	2.57 (1.83,3.4)	3.99 (2.82,5.25)	1.35 (0.95,1.81)
South Korea	2505 (1632,3623)	1850 (1200,2714)	654 (394,945)	7.74 (5.01,11.21)	12.81 (8.32,18.7)	3.85 (2.36,5.62)	4675 (3265,6678)	3436 (2337,4840)	1239 (829,1814)	5.04 (3.54,7.2)	8.01 (5.47,11.29)	2.41 (1.63,3.49)
Mongolia	152 (96,232)	95 (58,149)	57 (35,92)	12.35 (7.7,19.56)	16.98 (10.4,26.69)	8.46 (5.26,13.91)	362 (240,538)	204 (132,310)	158 (100,236)	15.17 (9.97,22.1)	18.4 (11.9,27.93)	12.49 (8.08,18.45)
