# Supplementary material for: The Burden of Liver Cancer in Selected East Asian Countries (1990–2021) and Projections up to 2036: A Systematic Analysis of the Global Burden of Disease Study 2021
Source: Cancers (Basel). 2026 Apr 16;18(8):1272. doi: 10.3390/cancers18081272 (PMC13115021; doi:10.3390/cancers18081272)
Supplement: Supplementary file 1 [file cancers-18-01272-s001.zip › cancers-4172898-supplementary/Table S5 YLLs.pdf]

Table S5. YLLs (Years of Life Lost) from 1990 to 2021 at the global, regional, and selected East Asian countries levels.

**Location**	**1990 YLLs cases (95% UI)**			**1990 Age-standardized rates per 100 000 people (95% UI)**			**2021 YLLs cases (95% UI)**			**2021 Age-standardized rates per 100 000 people (95% UI)**		
	**Total**	**Male**	**Female**	**Total**	**Male**	**Female**	**Total**	**Male**	**Female**	**Total**	**Male**	**Female**
Global	7495025 (6838235, 8227943)	5302419 (4720239, 5878994)	2192607 (1953232, 2466856)	171.47 (156.33,188.75)	249.6 (222.88,276.7)	96.93 (86.19,109.58)	12761152 (11560541, 14332217)	8988815 (7898898,10567197)	3772337 (3420654, 4157877)	147.81 (133.89,165.79)	215.51 (189.81,252.87)	84.19 (76.4,92.75)
SDI High SDI	1486280 (1396992, 1579787)	1103304 (1035521, 1179016)	382976 (352974,408519)	141.78 (133.27,150.93)	228.97 (214.92,244.6)	64.93 (60.08,69.16)	2417832 (2258054,2546751)	1708707 (1620198,1792601)	709125 (627180,757283)	126.16 (118.96,132.52)	190.16 (180.71,199.97)	67.3 (61.24,71.03)
High-middle SDI	1896635 (1657255, 2155442)	1401178 (1193752, 1639405)	495457 (428816,572486)	182.98 (160.2,207.6)	286.02 (244.82,332.89)	90.07 (77.95,103.93)	2849717 (2425074,3392751)	2109290 (1723584,2649545)	740427 (619215,885247)	151.14 (128.38,179.96)	235.48 (192.78,295.04)	72.25 (60.63,85.97)

Middle SDI	2700704 (2367718,3040512)	1927592 (1650360,2247462)	773112 (666670,881073)	215.17 (188.77,241.49)	302.35 (259.17,352.54)	126.32 (108.64,144.32)	4703780 (4077062,5599885)	3445759 (2865931,4299377)	1258021 (1084947,1471046)	168.17 (146.22,199.26)	251.14 (209.52,313.27)	88.76 (76.84,103.54)
Low-middle SDI	841909 (739780,992872)	526096 (457211,626591)	315813 (268992,383564)	111.87 (96.79,136.29)	137.28 (118.6,167.34)	85.19 (70.05,108.21)	1825379 (1637606,2033002)	1162455 (1012501,1340069)	662924 (595718,735928)	115.45 (104.01,128.21)	150.16 (131.37,173.22)	82.43 (73.99,91.41)
Low SDI	565175 (427477,722159)	341501 (240717,439132)	223674 (173990,301612)	192.06 (141.72,254.04)	233.55 (163.5,310.23)	149.04 (111.91,212.08)	957517 (766669,1234661)	557894 (439058,733441)	399623 (319638,510396)	149.87 (121.43,191.13)	176.09 (140.35,229.25)	124.09 (100.35,156.66)
Asia	5530507 (4934768,6108781)	4090005 (3567818,4620158)	1440502 (1257444,1619746)	231.67 (207.37,254.75)	336.22 (294.1,378.34)	124.13 (108.32,139.85)	8784743 (7657665,10223647)	6430124 (5402251,7948245)	2354618 (2056112,2711381)	169.76 (148.61,196.85)	251.42 (211.75,309.17)	90.23 (78.92,103.62)
China	3271883 (2745412,3849111)	2445505 (1986977,2995193)	826378 (662406,1002140)	332.05 (279.18,389.6)	480.48 (389.54,587.65)	176.05 (141.9,212.8)	4843420 (3867971,6072034)	3667951 (2778593,4930847)	1175469 (913934,1496493)	237.63 (190.4,296.93)	364.78 (277.17,486.49)	110.74 (86.25,140.35)
Japan	525140 (505658,537673)	412457 (401040,422167)	112683 (104396,116821)	304.24 (292.7,311.45)	516.46 (501.23,528.78)	118.64 (110.33,122.92)	491320 (437194,524048)	344791 (320502,360270)	146530 (113845,165369)	143.17 (131.67,150.45)	228.81 (216,238.09)	67.06 (56.72,72.97)
South Korea	337570 (250902,427529)	261291 (192298,339347)	76279 (54008,97657)	978.37 (735.7,1230.74)	1662.52 (1229.25,2122.12)	425.51 (301.88,542.23)	321661 (263008,397834)	251902 (205036,308382)	69759 (54680,86035)	349.53 (287.05,429.46)	575.88 (472.89,703.57)	140.22 (111.47,172.39)

Mongolia	21363 (15119,29260)	13423 (9098,18730)	7940 (5537,11098)	1701.14 (1200.91, 2332.24)	2323.42 (1585.67, 3294.57)	1161.73 (800.25,1620.39)	50909 (38857,65780)	29974 (22343,39096)	20935 (16139,27212)	1978.07 (1520.9,2528.38)	2491.29 (1866.05, 3236.09)	1549.32 (1193.6,2024.06)
