# Supplementary material for: The Burden of Liver Cancer in Selected East Asian Countries (1990–2021) and Projections up to 2036: A Systematic Analysis of the Global Burden of Disease Study 2021
Source: Cancers (Basel). 2026 Apr 16;18(8):1272. doi: 10.3390/cancers18081272 (PMC13115021; doi:10.3390/cancers18081272)
Supplement: Supplementary file 1 [file cancers-18-01272-s001.zip › cancers-4172898-supplementary/Table S6 DALYs.pdf]

Table S6. DALYs (Disability-Adjusted Life Years) from 1990 to 2021 at the global, regional, and selected East Asian countries levels.

Location	1990 DALYs cases (95% UI)			1990 Age-standardized rates per 100 000 people (95% UI)			2021 DALYs cases (95% UI)			2021 Age-standardized rates per 100 000 people (95% UI)		
	Total	Male	Female	Total	Male	Female	Total	Male	Female	Total	Male	Female
Global SDI	7553667 (6897511, 8296182)	5342652 (4758726, 5918719)	2211014 (1969875, 2485087)	172.86 (157.84, 190.16)	251.59 (224.76,278.87)	97.76 (86.85,110.42)	12887652 (11673533, 14472228)	9076177 (7970743,10670814)	3811476 (3455663, 4199822)	149.29 (135.24,167.48)	217.65 (191.58,255.35)	85.06 (77.25,93.7)
High SDI	1500946 (1410509, 1595214)	1113705 (1044598, 1191192)	387241 (357126,412853)	143.15 (134.48, 152.37)	231.15 (216.82,247.04)	65.62 (60.78,69.88)	2453150 (2289463,2587416)	1733197 (1643590,1821466)	719953 (636983,768555)	127.92 (120.57,134.41)	192.82 (183.1,202.84)	68.25 (62.22,71.98)
High-middle SDI	1910911 (1671066, 2172672)	1411349 (1203922, 1650323)	499562 (432250,576366)	184.38 (161.58, 209.2)	288.18 (246.92,335.28)	90.82 (78.57,104.67)	2877560 (2446560,3427884)	2129282 (1738603,2673351)	748278 (626072,895448)	152.6 (129.49,181.81)	237.73 (194.37,298.03)	72.99 (61.19,86.96)

Middle SDI	2719979 (2384909,3065584)	1940932 (1662468,2265147)	779047 (671211,887836)	216.8 (190.15,243.65)	304.59 (261.21,354.95)	127.36 (109.4,145.54)	4746028 (4107710,5643718)	3475834 (2890566,4335402)	1270194 (1095521,1485065)	169.71 (147.41,200.85)	253.4 (211.31,316.07)	89.63 (77.51,104.64)
Low-middle SDI	848140 (745555,1001884)	529930 (460467,631244)	318210 (271281,386622)	112.75 (97.58,137.45)	138.34 (119.5,168.84)	85.88 (70.62,109.02)	1839382 (1651209,2045500)	1171219 (1019886,1352238)	668163 (600498,740408)	116.37 (104.91,128.97)	151.35 (132.39,174.69)	83.11 (74.57,92.09)
Low SDI	569334 (430546,728123)	343967 (242443,442420)	225367 (175235,303887)	193.56 (142.76,255.75)	235.33 (164.9,312.49)	150.25 (112.76,213.8)	964542 (772939,1243934)	561891 (442820,738137)	402650 (321940,514583)	151.07 (122.38,192.78)	177.46 (141.62,231.14)	125.12 (101.05,157.96)
Asia	5572313 (4970859,6151703)	4119990 (3586772,4651782)	1452323 (1268682,1632881)	233.53 (208.97,256.87)	338.86 (295.94,381.25)	125.22 (109.31,140.97)	8871216 (7734221,10317636)	6491444 (5455877,8018269)	2379771 (2078943,2740928)	171.47 (150.05,198.86)	253.92 (214.16,312)	91.21 (79.78,104.74)
China	3294864 (2763029,3879589)	2462152 (2000446,3012119)	832712 (666725,1010058)	334.52 (281.08,393.14)	483.97 (392.36,591.53)	177.47 (142.84,214.58)	4890023 (3905089,6124599)	3702093 (2805347,4985654)	1187930 (924053,1513173)	239.91 (191.98,299.37)	368.19 (279.67,490.95)	111.91 (87.16,141.96)
Japan	531120 (511158,543692)	416875 (405198,426793)	114244 (105863,118540)	307.72 (295.94,315.01)	522.13 (506.88,534.75)	120.27 (111.81,124.65)	501032 (445194,534862)	351281 (326745,367518)	149752 (116034,169178)	145.74 (133.93,153.13)	232.8 (219.42,242.54)	68.41 (57.7,74.64)
South Korea	340074 (252485,430554)	263141 (193481,341442)	76933 (54472,98493)	986.11 (741.6,1242.07)	1675.33 (1238.06,2138.91)	429.36 (304.61,547.41)	326336 (267089,403348)	255338 (208125,312026)	70998 (55829,87946)	354.57 (291.34,435.31)	583.88 (478.97,712.66)	142.63 (113.69,176.14)

Mongolia	21514 (15232,29489)	13518 (9162,18843)	7997 (5580,11186)	1713.49 (1208.66,2349.1)	2340.4 (1596.81, 3318.08)	1170.19 (806.19, 1631.57)	51271 (39149,66245)	30178 (22481,39433)	21093 (16264,27452)	1993.25 (1531.38, 2547.25)	2509.69 (1880.45, 3262.38)	1561.8 (1204.42, 2043.09)
