# Supplementary material for: The Burden of Liver Cancer in Selected East Asian Countries (1990–2021) and Projections up to 2036: A Systematic Analysis of the Global Burden of Disease Study 2021
Source: Cancers (Basel). 2026 Apr 16;18(8):1272. doi: 10.3390/cancers18081272 (PMC13115021; doi:10.3390/cancers18081272)
Supplement: Supplementary file 1 [file cancers-18-01272-s001.zip › cancers-4172898-supplementary/Table S7 BAPC prediction ASIR.pdf]

Table S7. BAPC prediction ASIR in selected East Asian countries.

ASIR (95% UI)	China males	China females	Japan males	Japan females	South Korea males	South Korea females	Mongolia males	Mongolia females
2030	13.38 (9.36, 17.40)	4.22 (3.14, 5.30)	14.45 (11.12, 17.78)	5.04 (3.63, 6.45)	30.19 (25.04, 35.34)	8.56 (7.16, 9.96)	88.17 (64.82, 111.51)	50.08 (33.64, 66.52)
2036	12.32 (4.95, 19.69)	3.65 (1.81, 5.49)	13.98 (7.48, 20.48)	5.17 (2.25, 8.09)	28.78 (19.37, 38.19)	8.33 (5.76, 10.90)	84.28 (41.32, 127.25)	41.51 (15.12, 67.90)
