# Supplementary figures and images for: The Burden of Liver Cancer in Selected East Asian Countries (1990–2021) and Projections up to 2036: A Systematic Analysis of the Global Burden of Disease Study 2021
Source: Cancers (Basel). 2026 Apr 16;18(8):1272. doi: 10.3390/cancers18081272 (PMC13115021; doi:10.3390/cancers18081272)

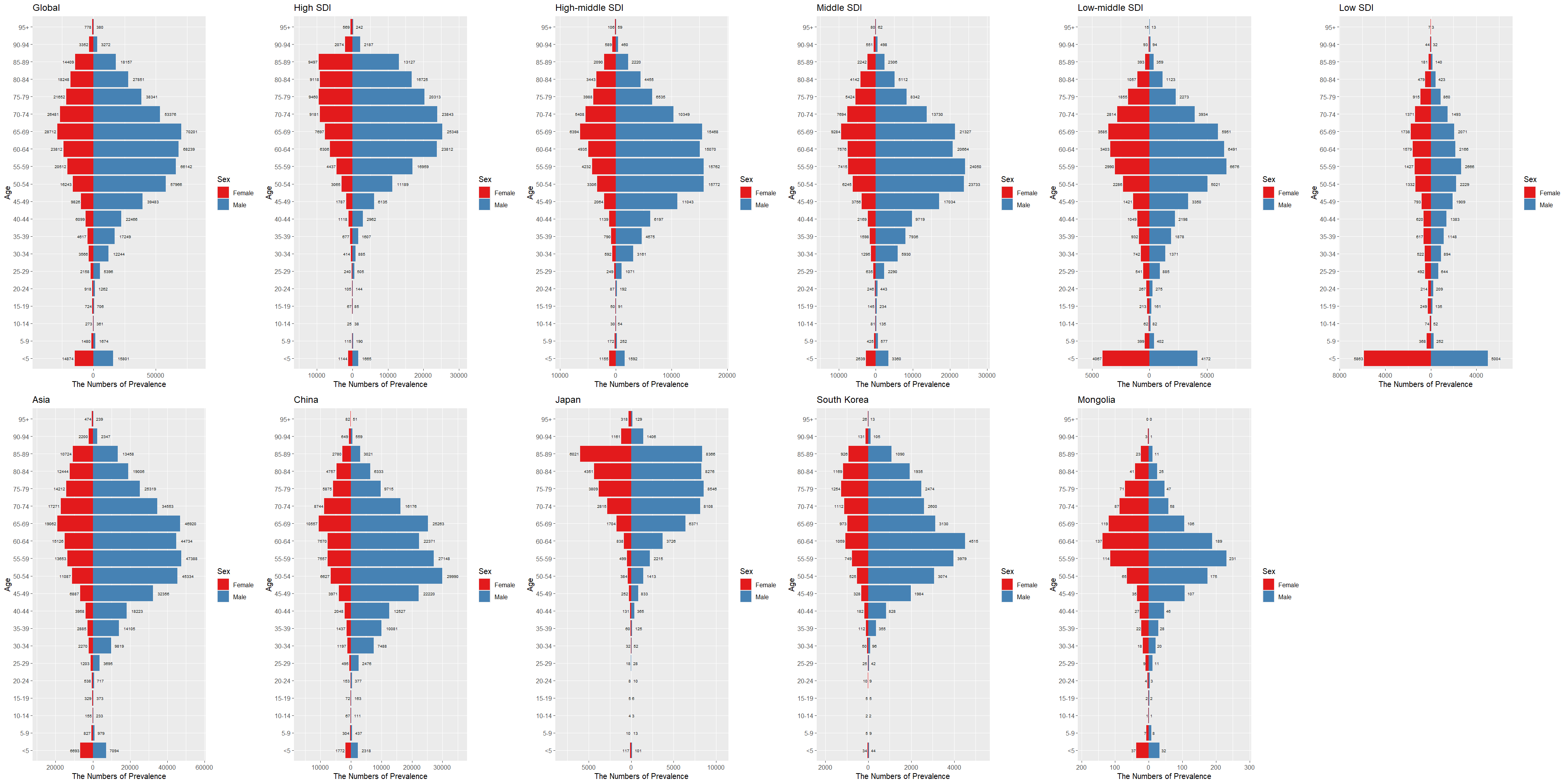

Supplement: Supplementary file 1 [file cancers-18-01272-s001.zip › cancers-4172898-supplementary/Figure S1 The Numbers of Prevalence.tiff]

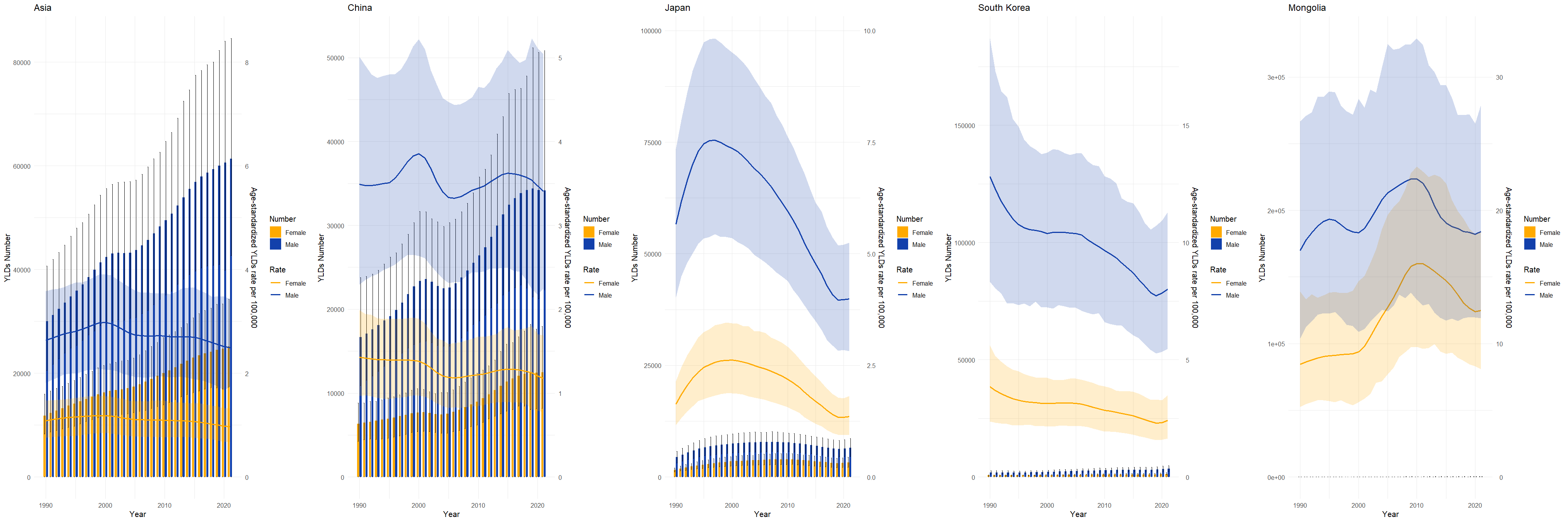

Supplement: Supplementary file 1 [file cancers-18-01272-s001.zip › cancers-4172898-supplementary/Figure S10 Dual-axis YLDs.tiff]

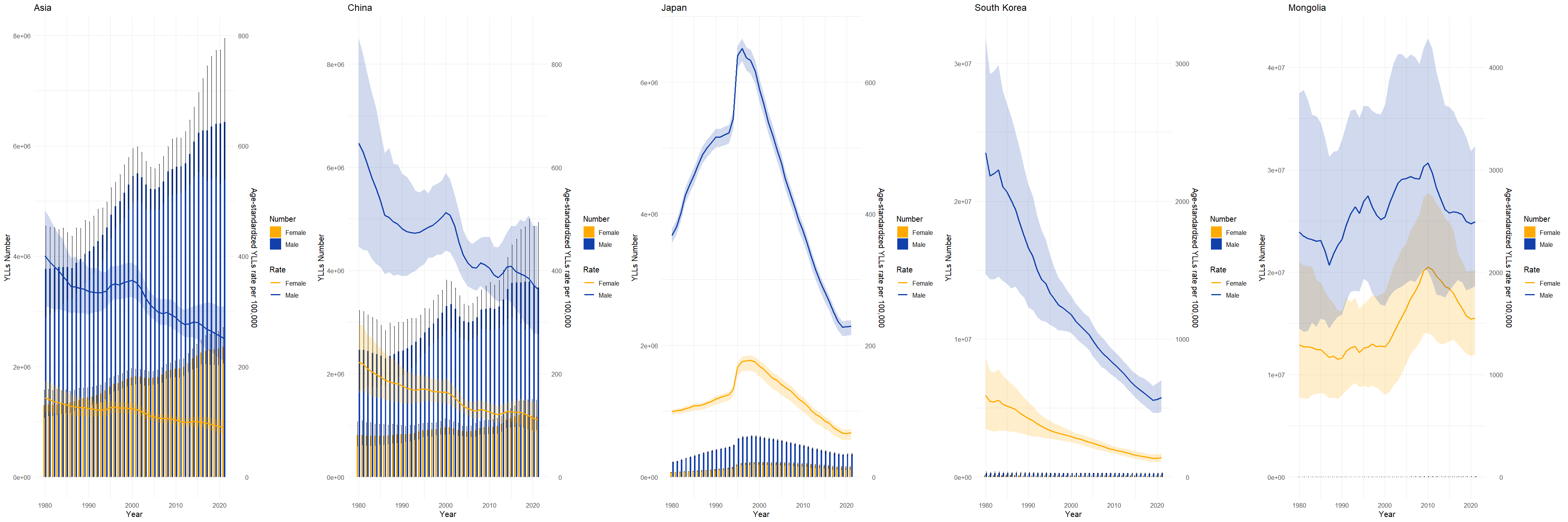

Supplement: Supplementary file 1 [file cancers-18-01272-s001.zip › cancers-4172898-supplementary/Figure S11 Dual-axis YLLs.tiff]

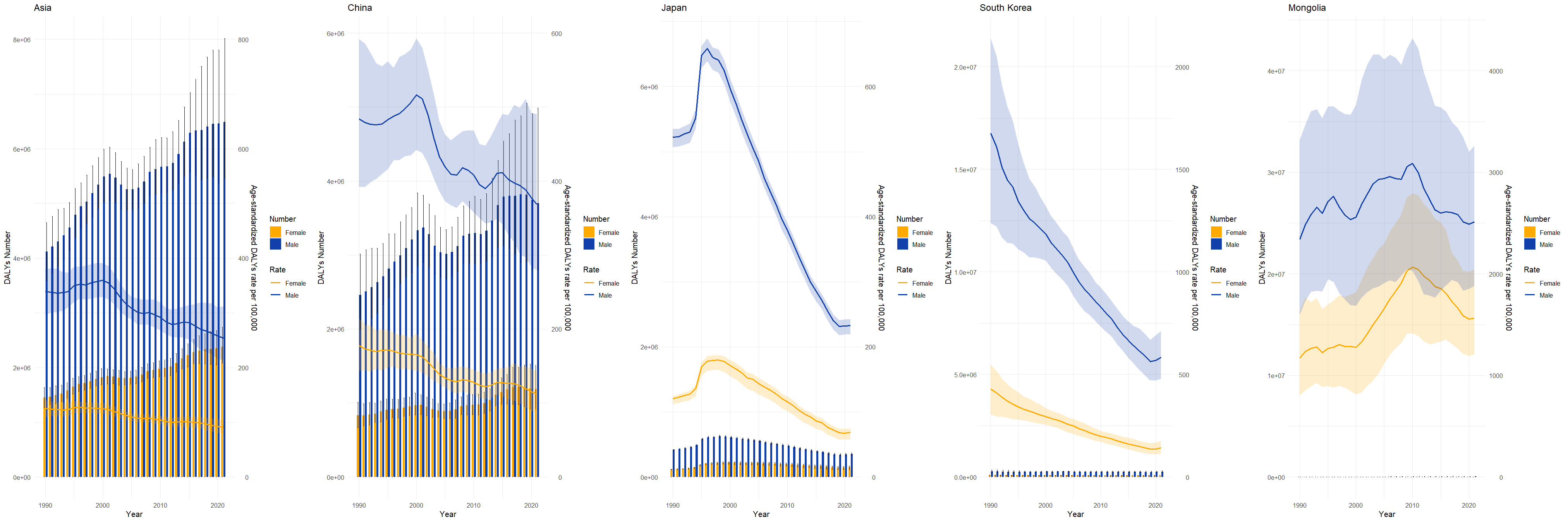

Supplement: Supplementary file 1 [file cancers-18-01272-s001.zip › cancers-4172898-supplementary/Figure S12 Dual-axis DALYs.tiff]

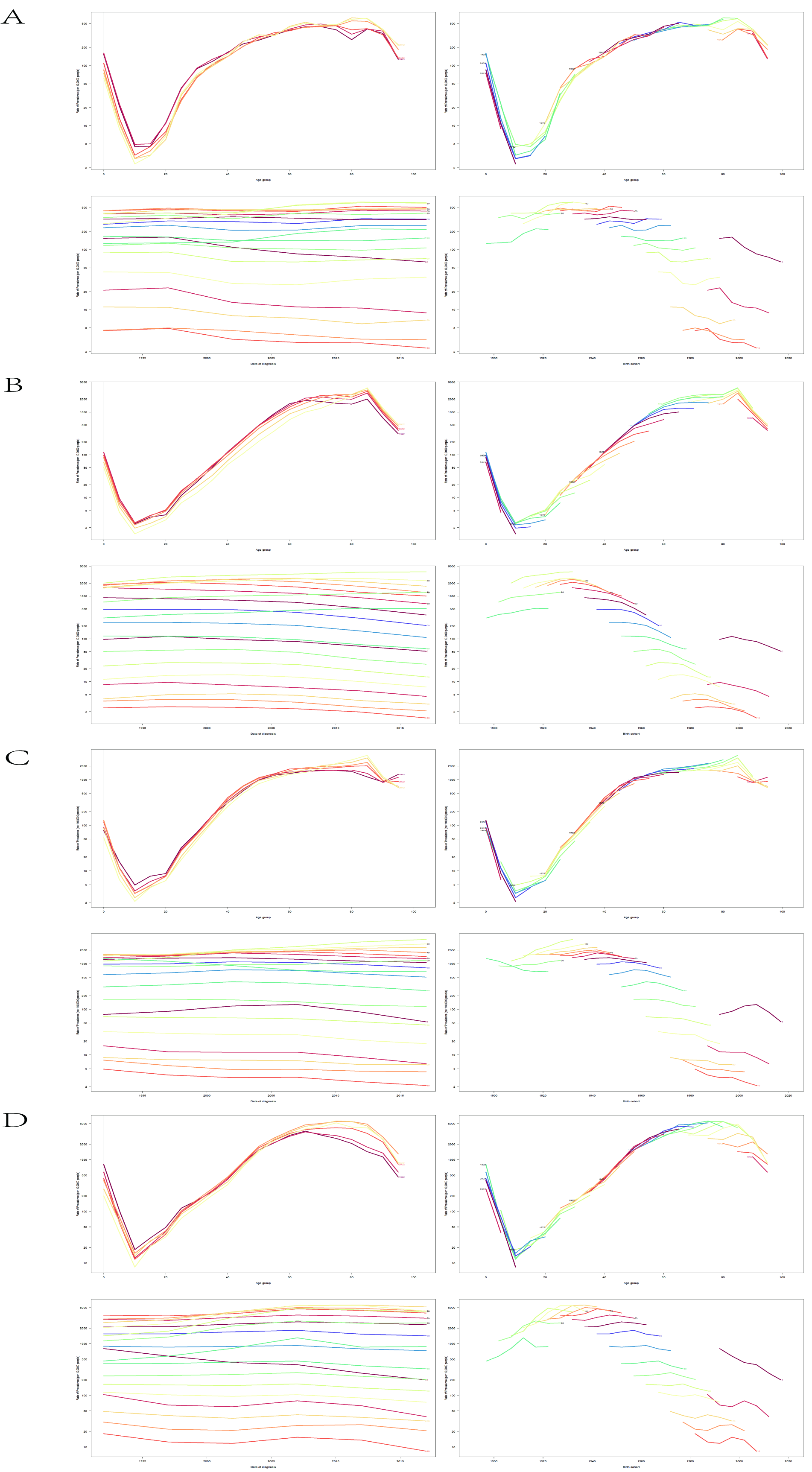

Supplement: Supplementary file 1 [file cancers-18-01272-s001.zip › cancers-4172898-supplementary/Figure S13.tif]

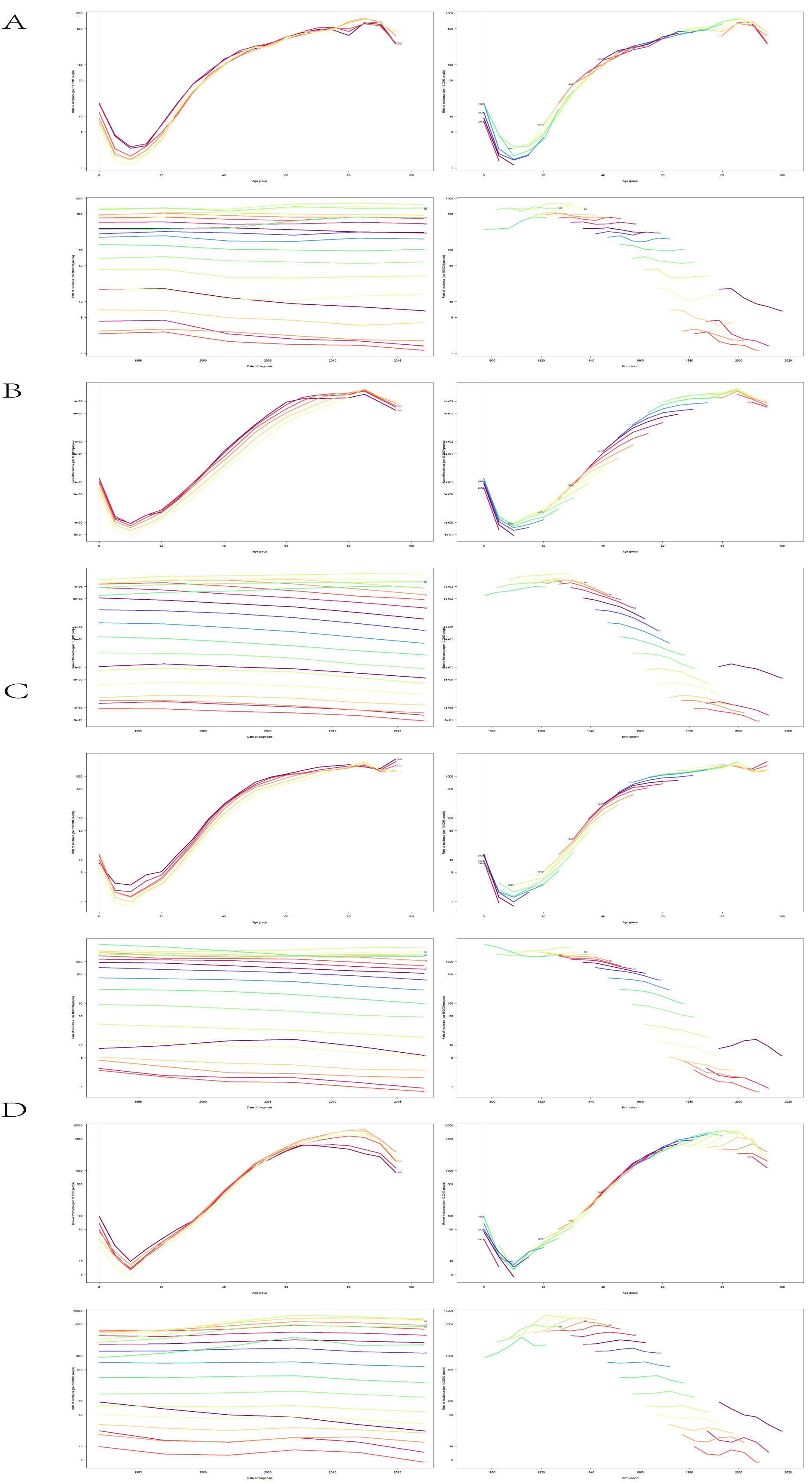

Supplement: Supplementary file 1 [file cancers-18-01272-s001.zip › cancers-4172898-supplementary/Figure S14.tif]

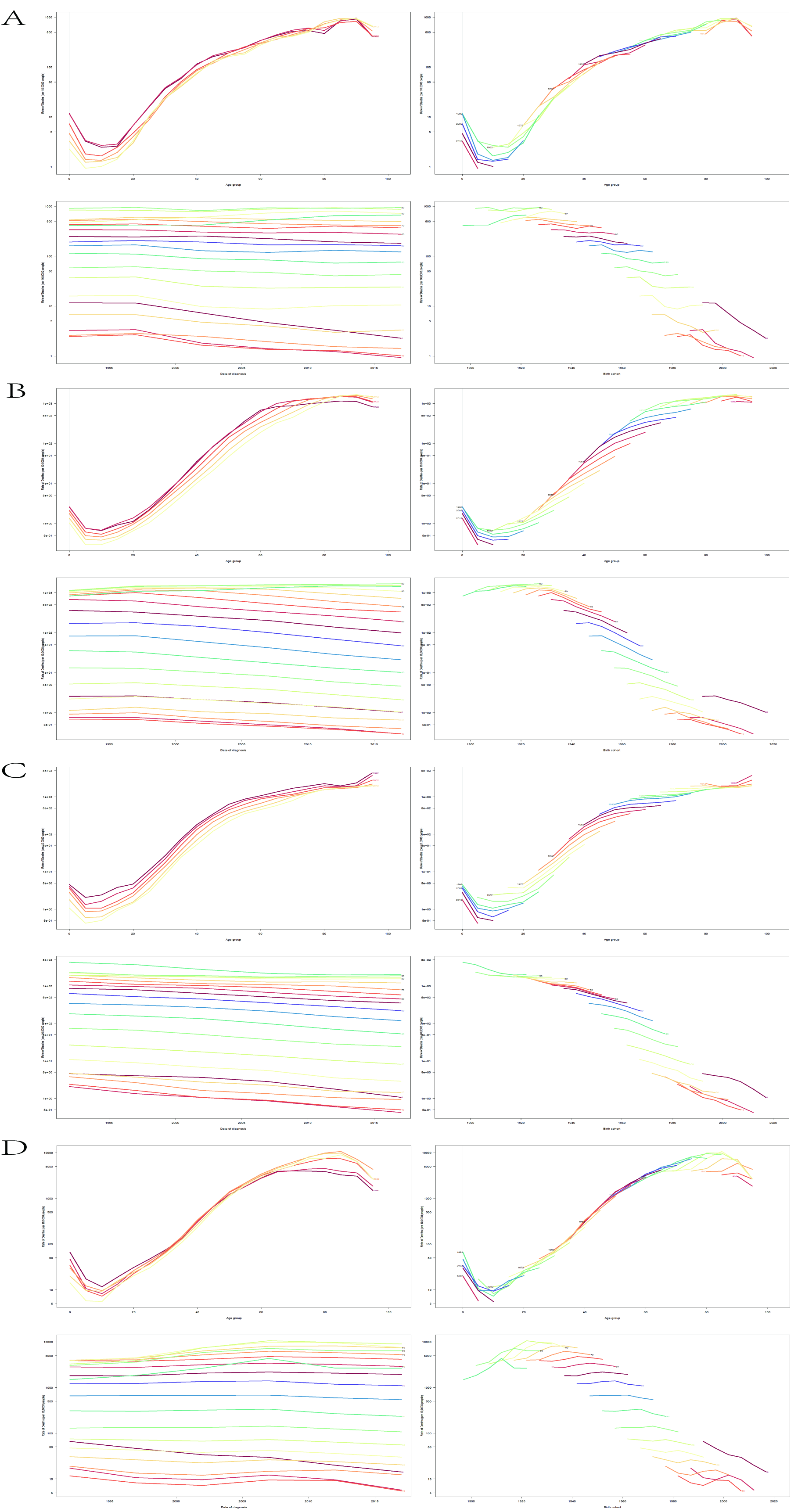

Supplement: Supplementary file 1 [file cancers-18-01272-s001.zip › cancers-4172898-supplementary/Figure S15.tif]

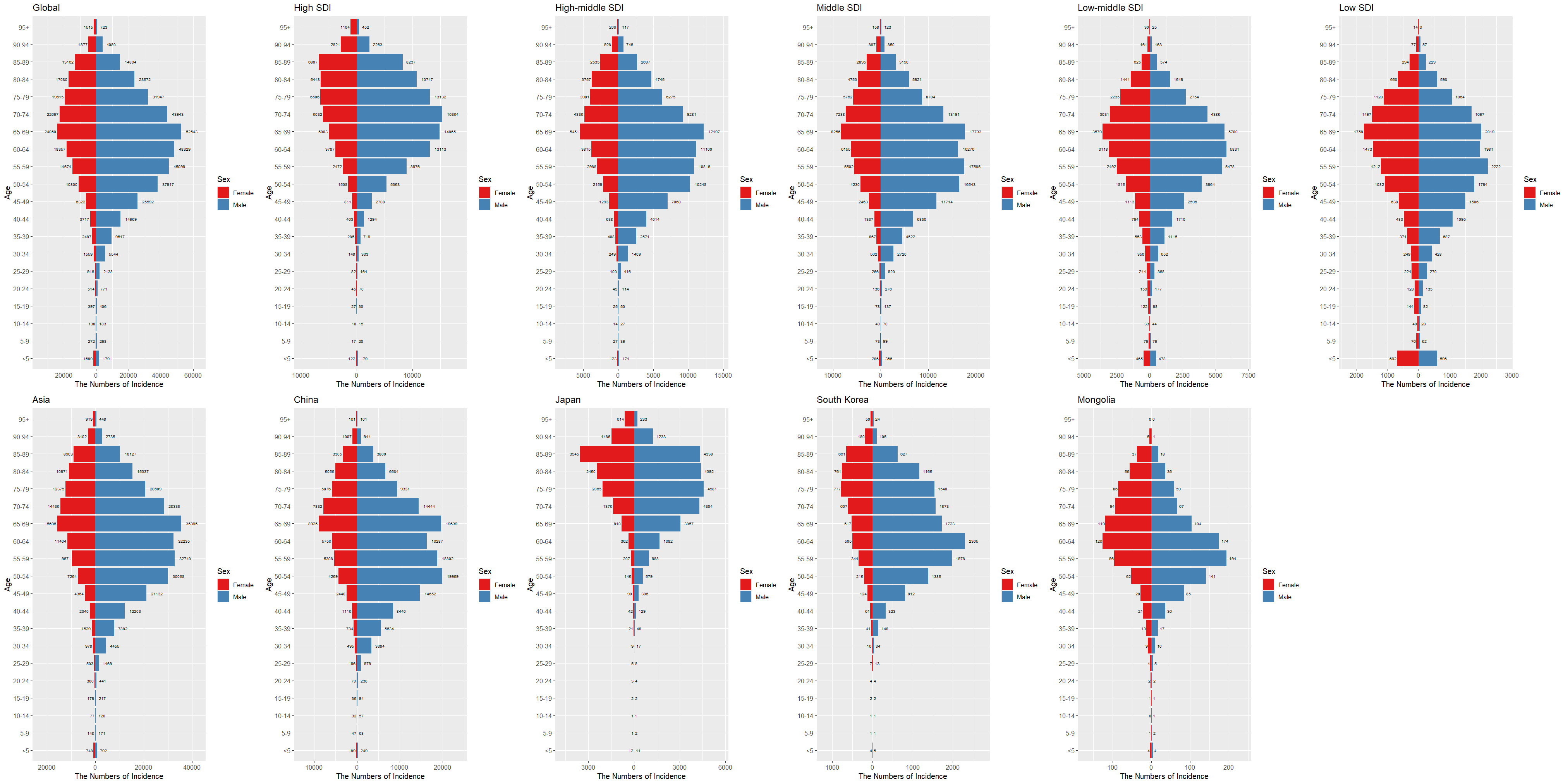

Supplement: Supplementary file 1 [file cancers-18-01272-s001.zip › cancers-4172898-supplementary/Figure S2 The Numbers of Incidence.tiff]

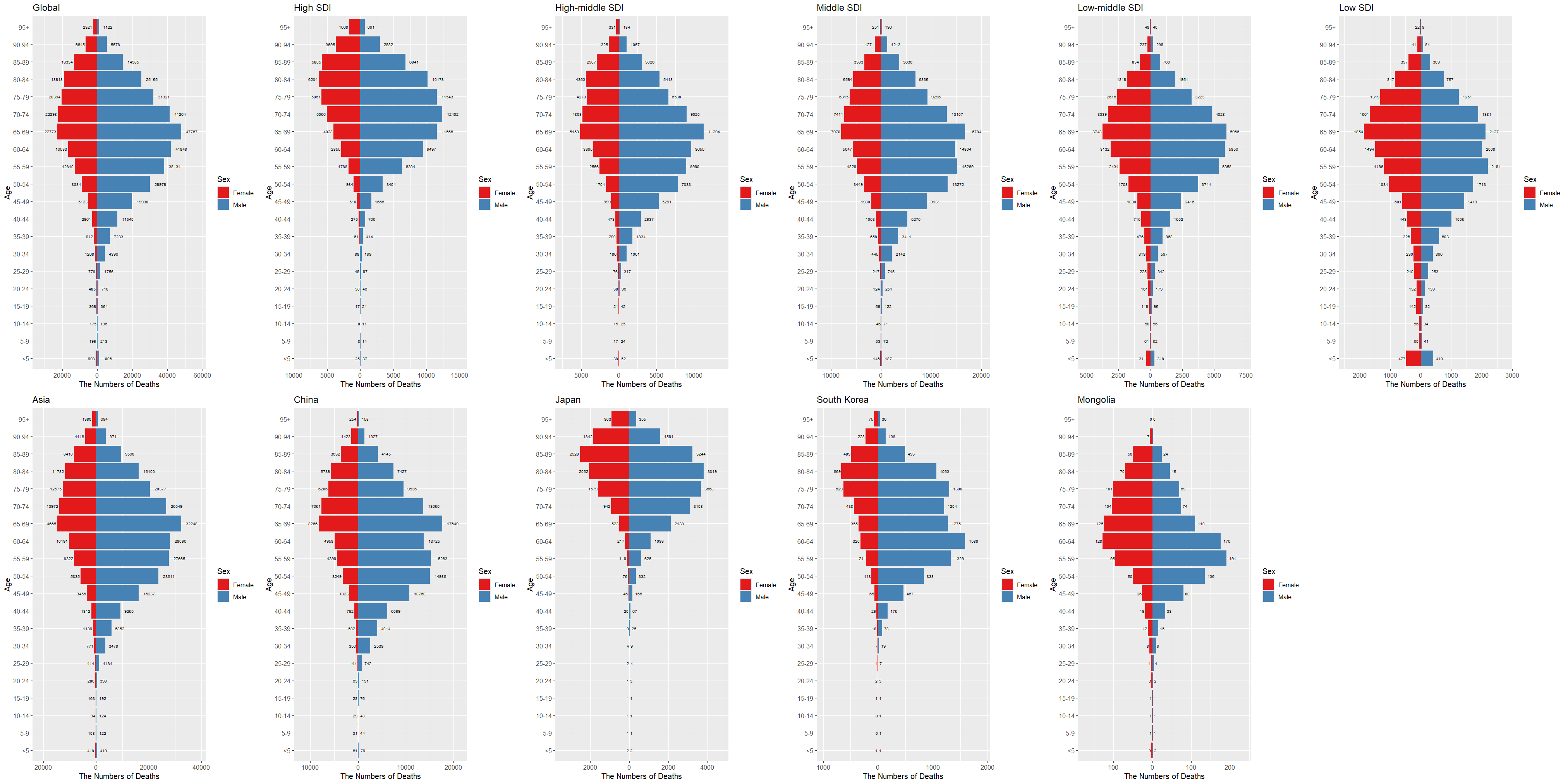

Supplement: Supplementary file 1 [file cancers-18-01272-s001.zip › cancers-4172898-supplementary/Figure S3 The Numbers of Deaths.tiff]

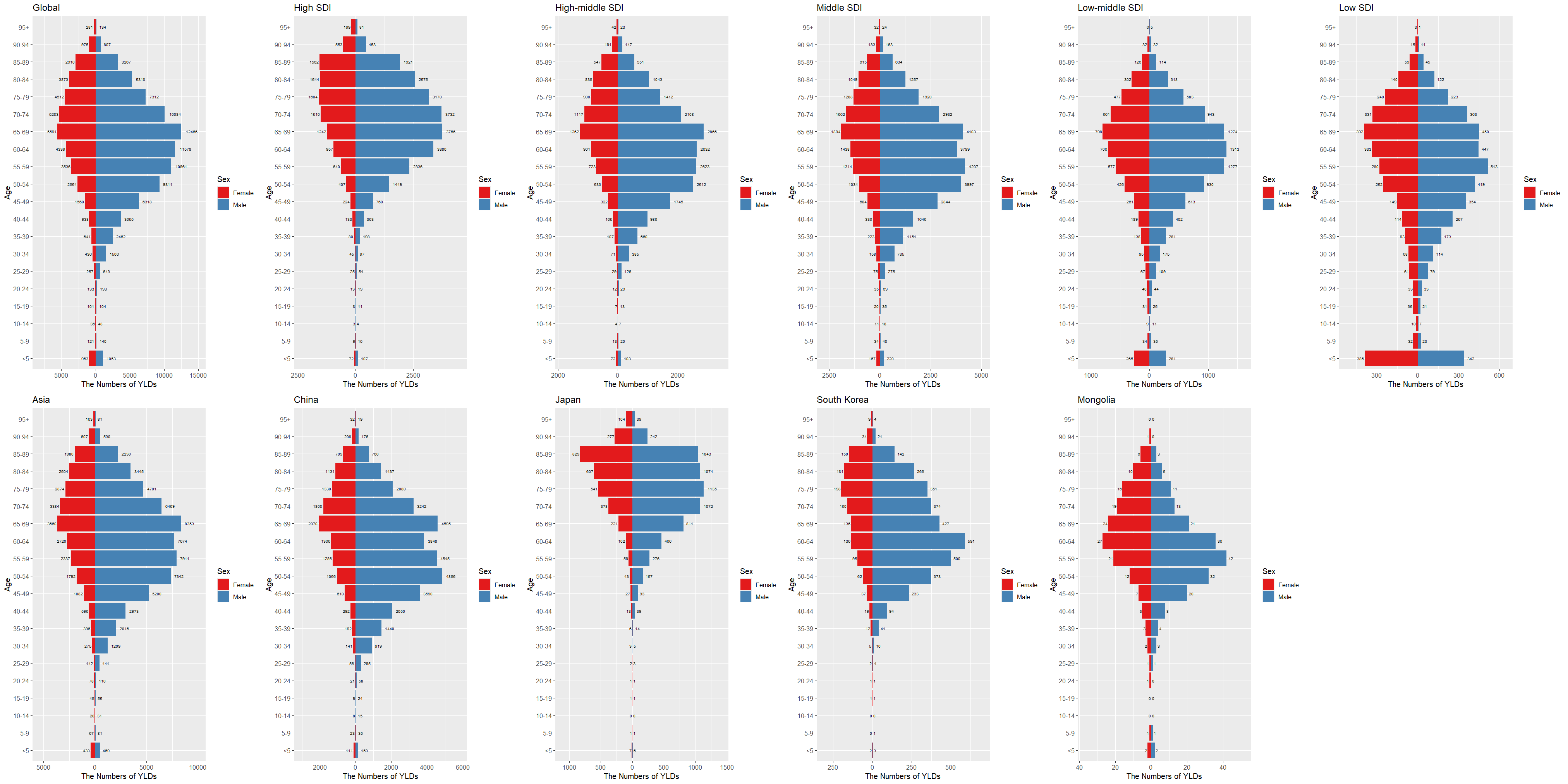

Supplement: Supplementary file 1 [file cancers-18-01272-s001.zip › cancers-4172898-supplementary/Figure S4 The Numbers of YLDs.tiff]

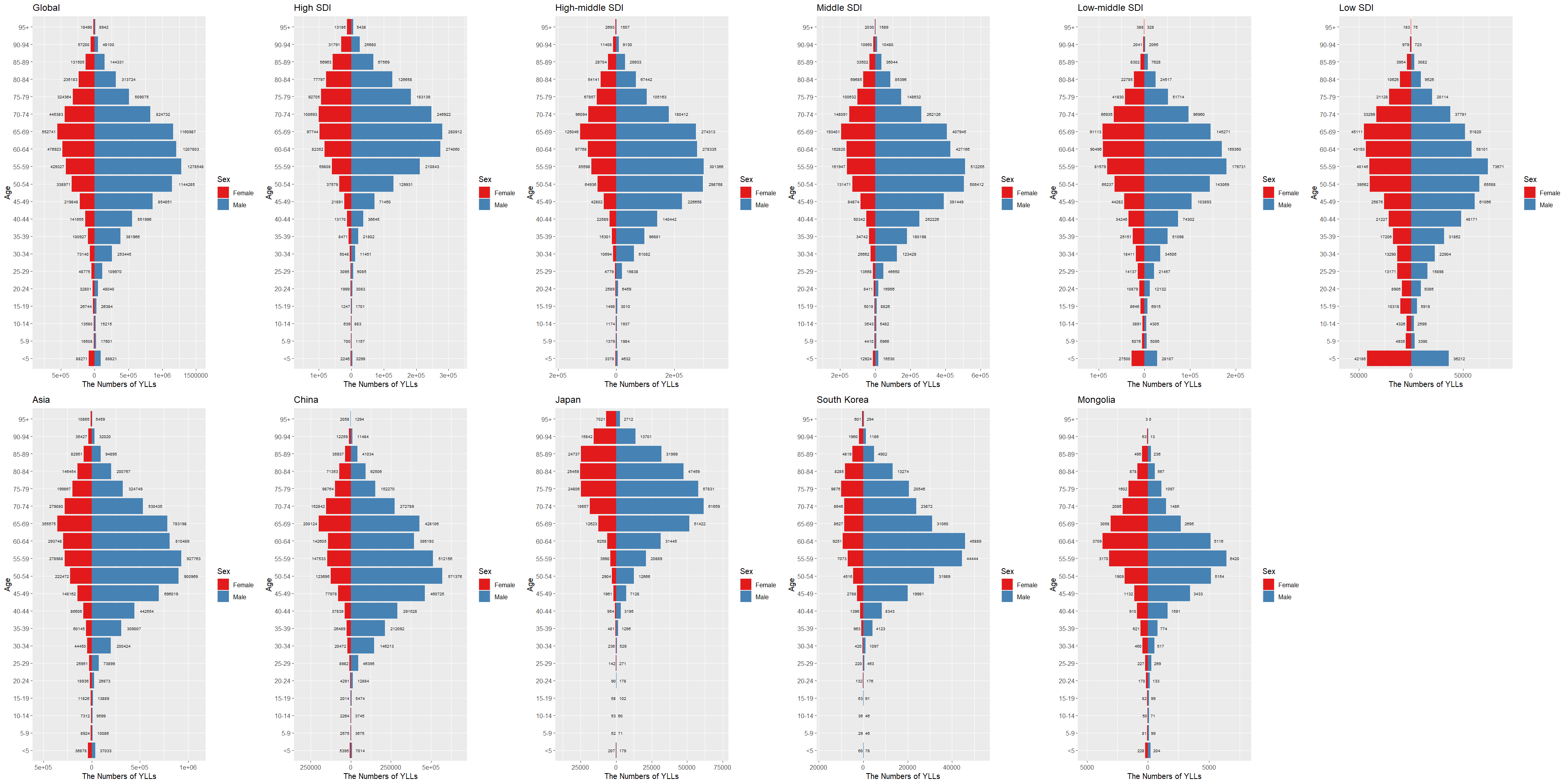

Supplement: Supplementary file 1 [file cancers-18-01272-s001.zip › cancers-4172898-supplementary/Figure S5 The Numbers of YLLs.tiff]

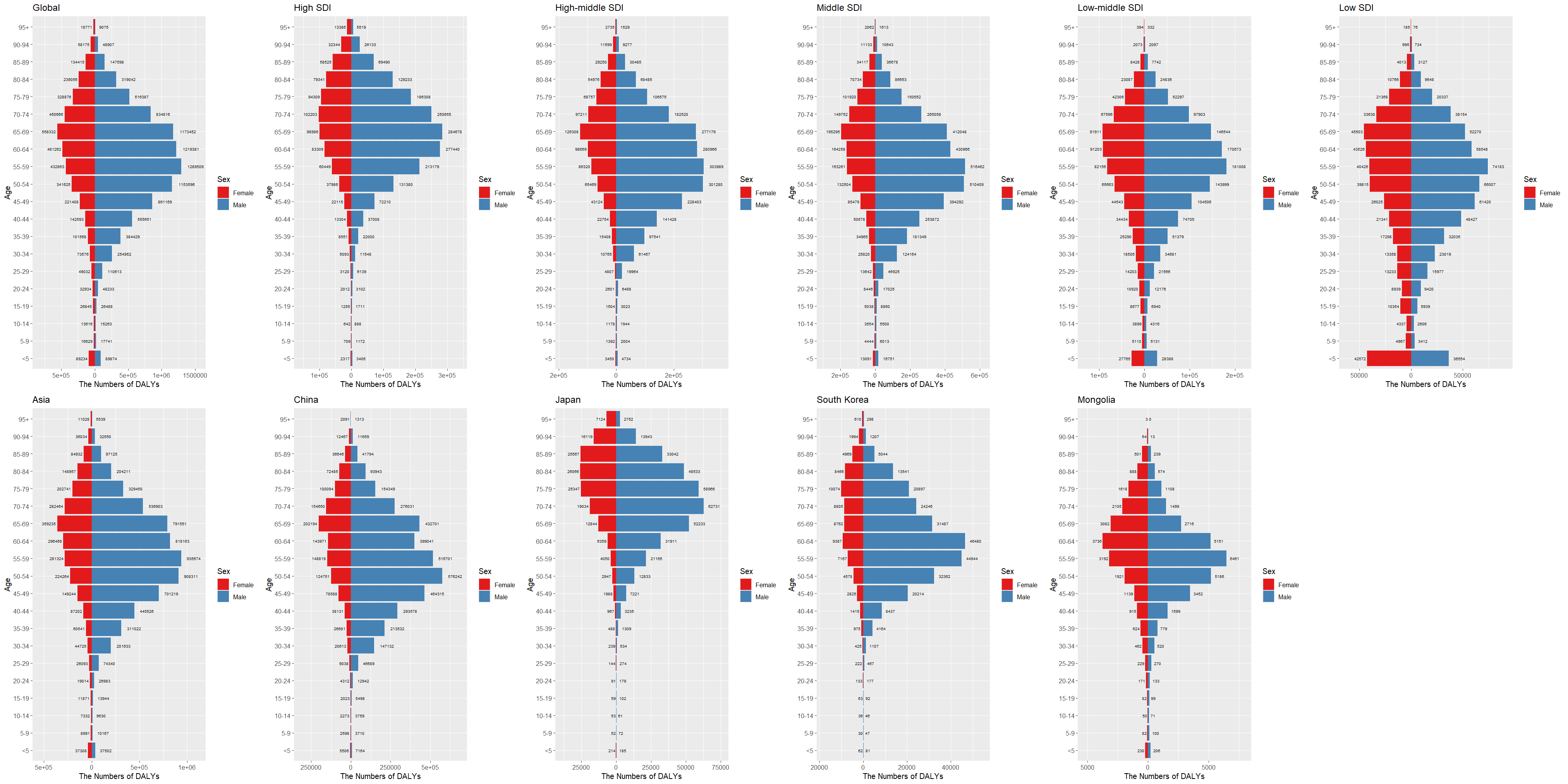

Supplement: Supplementary file 1 [file cancers-18-01272-s001.zip › cancers-4172898-supplementary/Figure S6 The Numbers of DALYs.tiff]

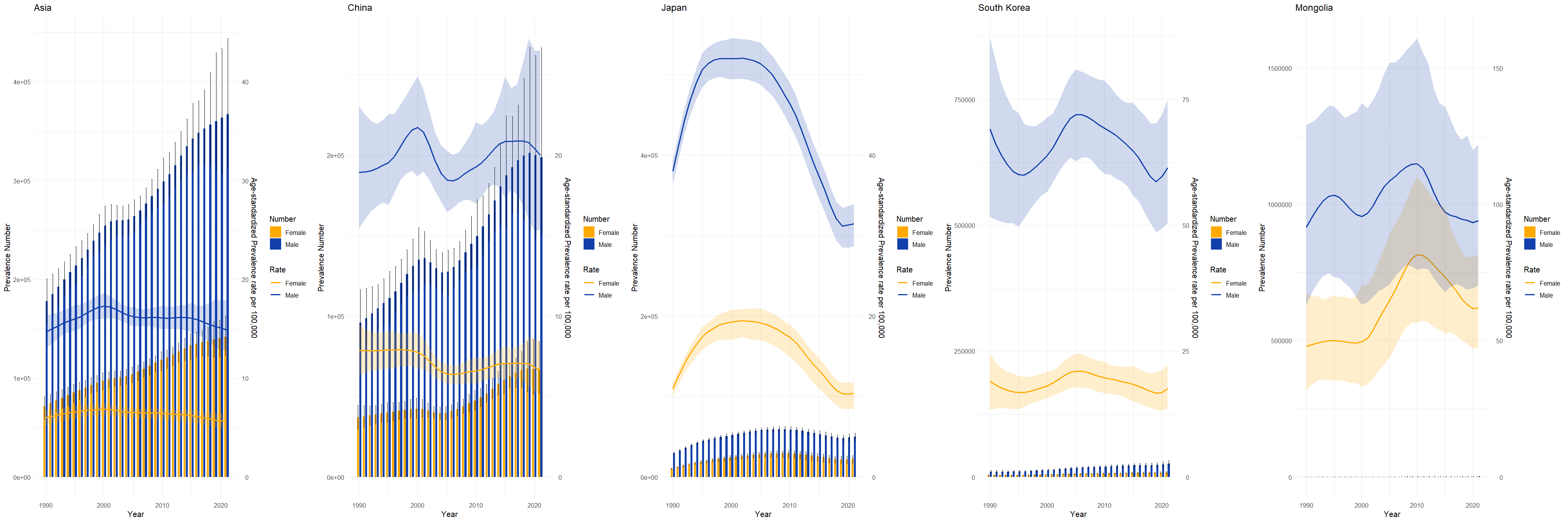

Supplement: Supplementary file 1 [file cancers-18-01272-s001.zip › cancers-4172898-supplementary/Figure S7 Dual-axis Prevalence.tiff]

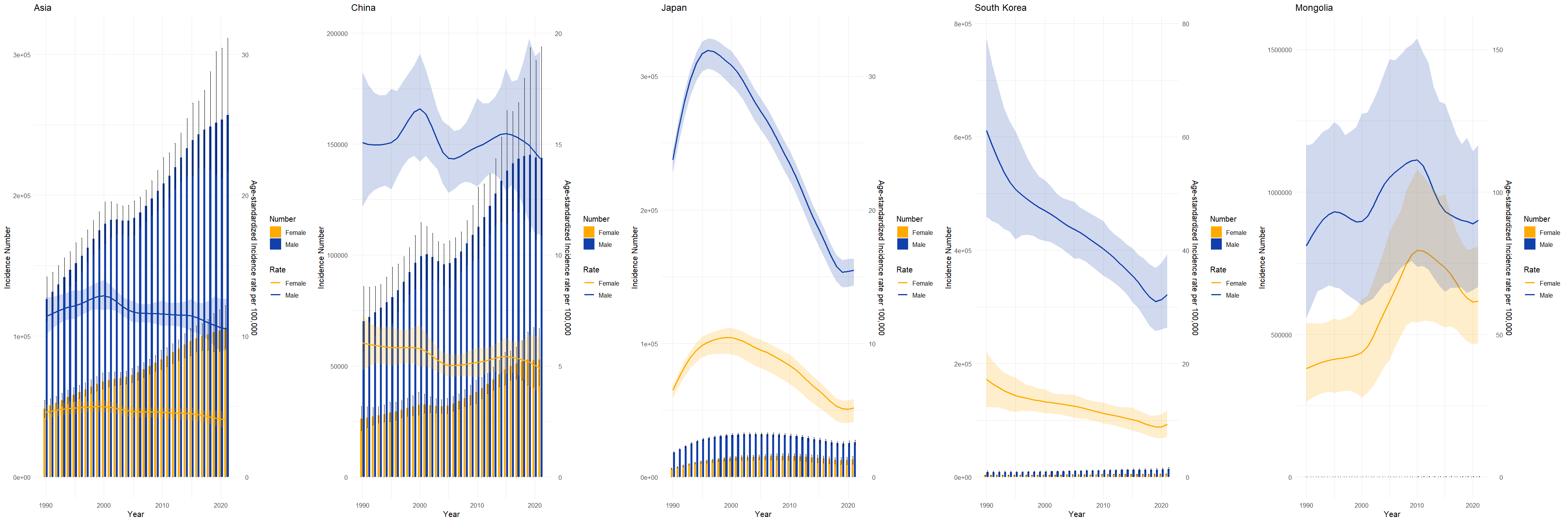

Supplement: Supplementary file 1 [file cancers-18-01272-s001.zip › cancers-4172898-supplementary/Figure S8 Dual-axis Incidence.tiff]

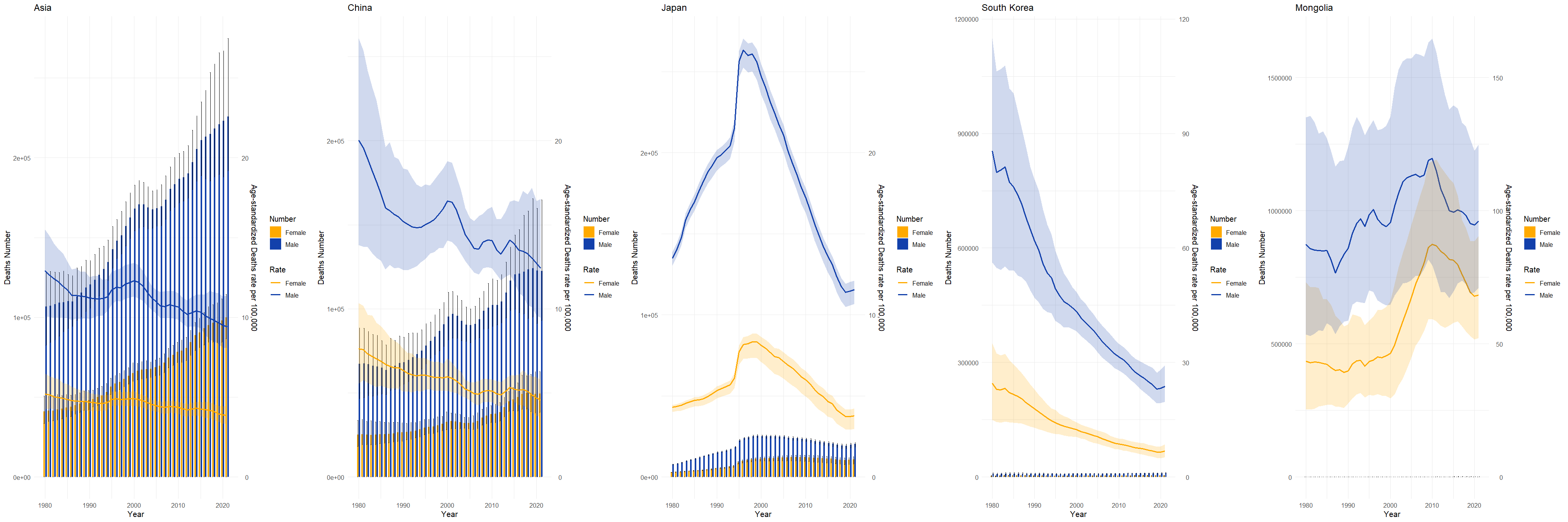

Supplement: Supplementary file 1 [file cancers-18-01272-s001.zip › cancers-4172898-supplementary/Figure S9 Dual-axis Deaths.tiff]
